# Elevated Kallistatin promotes the occurrence and progression of non-alcoholic fatty liver disease

**DOI:** 10.1038/s41392-024-01781-9

**Published:** 2024-03-12

**Authors:** Zhenzhen Fang, Gang Shen, Yina Wang, Fuyan Hong, Xiumei Tang, Yongcheng Zeng, Ting Zhang, Huanyi Liu, Yanmei Li, Jinhong Wang, Jing Zhang, Anton Gao, Weiwei Qi, Xia Yang, Ti Zhou, Guoquan Gao

**Affiliations:** 1 Department of Biochemistry, Zhongshan School of Medicine, Sun Yat-Sen University, Guangzhou, 510080 China; 2 Department of VIP Medical Center, the Third Affiliated Hospital of Sun Yat-Sen University, Guangzhou, 510080 China; 3 Physical Examination Center, the Third Affiliated Hospital of Sun Yat-Sen University, Guangzhou, 510080 China; 4 Department of Clinical Laboratory, Guangzhou First People’s Hospital, Guangzhou, 510080 China; 5 Guangdong Key Laboratory of Liver Disease Research, the Third Affiliated Hospital of Sun Yat-Sen University, Guangzhou, 510080 China; 6 Department of Health Sciences, College of Health Solutions, Arizona State University, Tempe, USA; 7 Guangdong Engineering & Technology Research Center for Gene Manipulation and Biomacromolecular Products, Sun Yat-Sen University, Guangzhou, 510080 China; 8 Guangdong Province Key Laboratory of Diabetology, Guangzhou, 510080 China; 9 Guangdong Province Key Laboratory of Brain Function and Disease, Zhongshan School of Medicine, Sun Yat-Sen University, Guangzhou, 510080 China

**Keywords:** Metabolic disorders, Molecular medicine

## Abstract

Non-alcoholic fatty liver disease (NAFLD) is the most common chronic liver disease worldwide, and the development of non-alcoholic steatohepatitis (NASH) might cause irreversible hepatic damage. Hyperlipidemia (HLP) is the leading risk factor for NAFLD. This study aims to illuminate the causative contributor and potential mechanism of Kallistatin (KAL) mediating HLP to NAFLD. 221 healthy control and 253 HLP subjects, 62 healthy control and 44 NAFLD subjects were enrolled. The plasma KAL was significantly elevated in HLP subjects, especially in hypertriglyceridemia (HTG) subjects, and positively correlated with liver injury. Further, KAL levels of NAFLD patients were significantly up-regulated. KAL transgenic mice induced hepatic steatosis, inflammation, and fibrosis with time and accelerated inflammation development in high-fat diet (HFD) mice. In contrast, KAL knockout ameliorated steatosis and inflammation in high-fructose diet (HFruD) and methionine and choline-deficient (MCD) diet-induced NAFLD rats. Mechanistically, KAL induced hepatic steatosis and NASH by down-regulating adipose triglyceride lipase (ATGL) and comparative gene identification 58 (CGI-58) by LRP6/Gɑs/PKA/GSK3β pathway through down-regulating peroxisome proliferator-activated receptor γ (PPARγ) and up-regulating kruppel-like factor four (KLF4), respectively. CGI-58 is bound to NF-κB p65 in the cytoplasm, and diminishing CGI-58 facilitated p65 nuclear translocation and TNFα induction. Meanwhile, hepatic CGI-58-overexpress reverses NASH in KAL transgenic mice. Further, free fatty acids up-regulated KAL against thyroid hormone in hepatocytes. Moreover, Fenofibrate, one triglyceride-lowering drug, could reverse hepatic steatosis by down-regulating KAL. These results demonstrate that elevated KAL plays a crucial role in the development of HLP to NAFLD and may be served as a potential preventive and therapeutic target.

## Introduction

NAFLD also known as metabolic dysfunction-associated steatotic liver disease (MASLD), is a prevalent liver disease whose prevalence is as high as 25%,^1^ characterized by excessive triglyceride (TG) deposition in hepatocytes, excluding significant alcohol consumption and other damaging factors.^2^ A significant proportion of NAFLD patients in Asia are not obese; HLP, particularly HTG, was a significant risk factor for NAFLD;^3–5^ Further, it is reported that non-obese NAFLD usually has higher plasma TG levels when compared with matched controls without NAFLD.^6^ However, the linking molecules and mechanisms between HLP and NAFLD are not fully understood.

NAFLD is a general term for a series of liver-related diseases, including hepatic steatosis, NASH, and liver cirrhosis.^7^ An imbalance between TG synthesis and clearance would cause accumulation of lipid droplet-like TG in the liver and induce hepatic steatosis.^2^ Hepatic steatosis is often self-limited and reversible, but part of it can develop into NASH, which will cause irreversible damage to the liver, such as liver cirrhosis, liver failure, and hepatocellular carcinoma.^8^ In addition, NASH also causes non-liver-related adverse outcomes, such as cardiovascular disease and malignant tumors.^9^ However, there is currently no approved drug for NASH treatment.^9^ Knowledge of the pathogenesis of hepatic steatosis into NASH and fibrosis is still incomplete. Currently, chronic liver inflammation is knowledgeably considered to be the leading cause of NASH,^10^ and the NF-κB/TNFα signaling pathway is an important inflammatory signaling pathway involved in the development of chronic inflammation in NAFLD.^11^

KAL, encoded by the *SERPINA4*, is a secreted protein mainly expressed in hepatocytes.^12^ The previous studies, including ours’ showed that KAL levels in the peripheral blood of diabetic patients were significantly elevated^13,14^ and positively correlated with TG.^14,15^ Furthermore, we have reported that KAL could activate the nuclear factor κB (NF-κB) signaling pathway to cause inflammation in diabetic wound tissues.^13^ However, the relationship between lipid metabolic dysfunction, KAL, and NAFLD remains unclear. The Bioinformatic analysis of the plasma proteome also revealed several other significantly up-regulated proteins, IGHV4-4 (Immunoglobulin heavy variable 4-4), LAMB1 (Laminin subunit beta 1), and VNN1 (Vanin 1) (supplementary Fig. 1a). LAMB1 is a kind of extracellular matrix glycoprotein, which is mainly related to cell adhesion and migration; VNN1 is also a secreted protein mainly expressed in hepatocytes, however, it has been reported that knockout of VNN1 can promote the accumulation of TG in the liver under fasting state,^16^ suggesting that elevated VNN1 may not be a inducer of NAFLD. Thus, we choose SERPINA4 (KAL) for further study.

The present study aims to illuminate the role of KAL in the occurrence and development of NAFLD and the intermediary between HLP and NAFLD. Our results demonstrate for the first time that serum KAL is increased in NAFLD and acts as a potent initiator of hepatic steatosis and inflammation, promoting the progression of HFD mice from steatosis to NASH. Conversely, *Serpina4*^−/−^ alone had no effect on lipid metabolism and liver lesions, but ameliorated hepatic steatosis and inflammation in HFruD and MCD induced NAFLD rats. Mechanistically, KAL induced hepatic steatosis and NASH by down-regulating ATGL and CGI-58 by LRP6/Gɑs/PKA/GSK3β pathway. Consequently, diminishing CGI-58 activated NF-κB/TNFα signaling pathway. These data indicate that KAL is a critical regulatory molecule that mediates the occurrence and progression of NAFLD.

## Results

### The level of KAL is increased in HLP and NAFLD patients and positively correlated with TG

We reanalyzed a plasma proteome profiling in ProteomeXchange (PRIDE archive PXD011839, https://www.ebi.ac.uk/pride/archive/). The samples were divided into HTG and control groups based on TG levels (individuals with TG ≥ 1.7 mM are defined as HTG), excluding obese individuals and individuals over 60 years of age. With at least one valid value in the samples, we get a dataset of 593 protein groups. Using Student’s t-test to determine the significantly changed proteins with a p-value of 0.05. We found that KAL was significantly up-regulated in the HTG group (supplementary Fig. 1a).

**Fig. 1:**
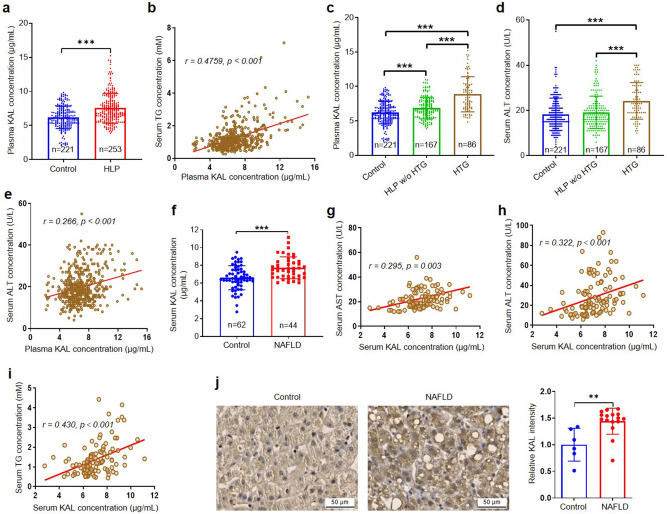
The content of KAL is increased in HLP and NAFLD patients, and positively correlated with TG. **a** The plasma KAL concentration in HLP subjects. **b** Correlation analysis of plasma KAL levels and TG concentrations. **c** The plasma KAL concentration in control and HLP subjects. **d** The serum ALT concentration in control and HLP subjects. **e** Correlation analysis of plasma KAL levels and ALT concentrations in control and HLP subjects. (**a**–**e**: Control, n = 221; HLP, n = 253; HLP w/o HTG, HLP without HTG, n = 167; HTG, n = 86). **f** The serum KAL concentration in NAFLD patients. **g**–**i** Correlation analysis of serum KAL levels with AST, ALT and TG. (**f**–**i**: Control, n = 62; NAFLD, n = 44). **j** Representative images and quantification of KAL level in the livers from NAFLD patients (Control, n = 6; NAFLD, n = 16). Scale bar: 50 μm. Data are expressed as mean ± SD. **p < *0.01*, ***p < 0.001

To further investigate the potential association between KAL and HLP, we conducted a study involving 253 HLP and 221 age-matched healthy controls. The clinical and biochemical characteristics of the participants are summarized in supplementary Table 1. Our results indicated that plasma KAL levels were significantly higher in HLP subjects compared to healthy control and positively correlated with TG, free fatty acid (FFA), total cholesterol (TC), low-density lipoprotein cholesterol (LDL-C), and negatively correlated with high-density lipoprotein cholesterol (HDL-C) (Fig. 1a-b, supplementary Fig. 1b-e). Moreover, the plasma KAL levels in HTG subjects were even higher than those in the HLP without HTG group (Fig. 1c). We also found that the alanine transaminase (ALT) and aspartate aminotransferase (AST) levels in HTG were higher than those in the HLP without HTG group and the healthy control group and positively correlated with KAL (Fig. 1d-e, supplementary Fig. 1f-g).

In addition, the serum KAL levels were higher in non-obese NAFLD patients than in matched healthy controls (Fig. 1f) and positively correlated with AST, ALT, and TG but not TC, LDL-C, and HDL-C (Fig. 1g-i, supplementary Fig. 1h-j, supplementary Table 2). Similarly, the KAL staining was increased in the liver of mild NAFLD patients (Fig. 1j). These findings suggested that elevated KAL levels are involved in the accumulation of TG and subsequent liver damage.

### Elevated KAL induces hepatic steatosis and NASH in chow-diet mice and aggravates hepatic steatosis to NASH in HFD mice

**Fig. 2:**
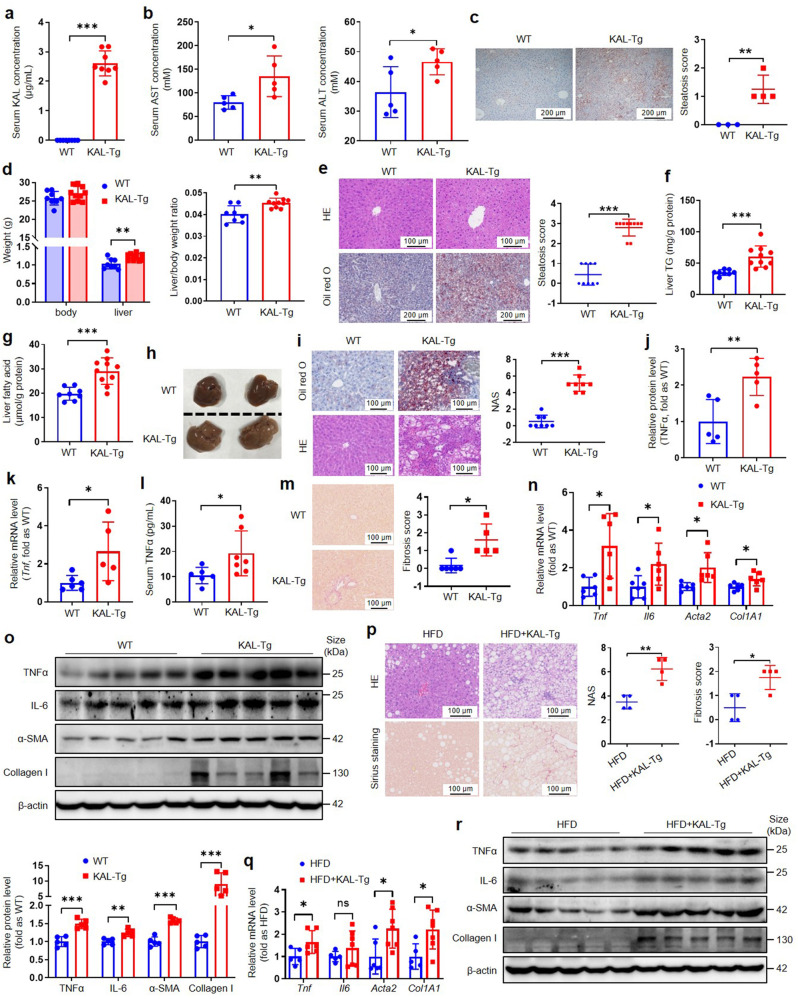
KAL induces hepatic steatosis and NASH in chow-diet mice and progresses hepatic steatosis to NASH in HFD mice. **a** The serum KAL concentration of KAL-Tg mice (n = 8). **b** The serum AST and ALT levels of 6-month-old mice (n = 5). **c** Oil red O staining and steatosis score of livers from 3-month-old mice (WT, n = 3; KAL-Tg, n = 4). Scale bar: 200 μm. **d**–**g** Weight, H&E (Scale bar: 100 μm) and Oil red O (Scale bar: 200 μm) staining, steatosis score, TG and fatty acid content of liver tissues from 6-month-old mice (n ≥ 8 per group). **h**–**l** Representative images, Oil red O and H&E staining, NAS, TNFα level of livers and serum TNFα in 10-month-old mice (n ≥ 5 per group). Scale bar: 100 μm. **m**–**o** Sirius staining and fibrosis scores, mRNA levels, and protein levels of genes related to inflammation and fibrosis in livers from 16-month-old mice (n ≥ 5 per group). Scale bar: 100 μm. **p**–**r** H&E and Sirius staining, NAS, fibrosis scores, mRNA levels and protein levels of genes related to inflammation and fibrosis of livers from mice fed an HFD for 28 weeks (n ≥ 4 per group). Scale bar: 100 μm. Data are expressed as mean ± SD. *p < 0.05, **p < 0.01, ***p < 0.001

To investigate the role of KAL in NAFLD, we conducted experiments on KAL-transgenic (KAL-Tg) mice. And the average serum KAL concentration of KAL-Tg mice was 2.6 μg/mL (Fig. 2a), which is of the same order of magnitude as that in the human HLP population (Fig. 1a, 2-10 μg/mL). We observed that KAL-Tg mice exhibited increased serum AST and ALT levels (Fig. 2b), indicating liver damage. Moreover, KAL-Tg mice exhibited slight hepatic lipid droplet deposition at 3 months and developed severe hepatic steatosis, disordered arrangement of hepatocytes, elevated hepatic TG and fatty acid levels, and increased liver weight at 6 months (Fig. 2c-g). It suggested that elevated KAL contributes to hepatic steatosis.

To further investigate the effect of KAL on NASH, we found that the livers of 10-month-old KAL-Tg mice exhibited yellow and granular surfaces, and developed NASH with apparent inflammation, balloon-like degeneration of hepatocytes, and NAFLD activity score (NAS) greater than 5 (Fig. 2h-i, supplementary Fig. 2a-d). The expression and secretion of the inflammatory factor tumor necrosis factor α (TNFα) were also increased (Fig. 2j-l, supplementary Fig. 2e). Since the 10-month-old mice did not exhibit extensive fibrosis with collagen deposition (supplementary Fig. 2f), we continued to raise the mice for 16-month-old and found that 16-month-old KAL-Tg mice developed not only NASH (supplementary Fig. 2g–i) but also hepatic fibrosis with elevated α-smooth muscle actin (α-SMA) and collagen I (Fig. 2m-o).

Additionally, to clarify whether KAL speeds up the development of hepatic steatosis to NASH, the classic model of hepatic steatosis, HFD mice, was used. When we fed KAL-Tg mice and wild-type (WT) mice with HFD, KAL-Tg mice exhibited extensive inflammation, injury, and fibrosis along with aggravated hepatic steatosis (Fig. 2p, supplementary Fig. 2j-m), and the expression of TNFα, interleukin-6 (IL-6), α-SMA, and collagen I was also increased (Fig. 2q-r, supplementary Fig. 2n). Our findings suggest that elevated KAL contributes to the progression of simple hepatic steatosis to NASH in HFD mice.

### Knockout of KAL ameliorates hepatic steatosis and inflammation in NAFLD rats

**Fig. 3:**
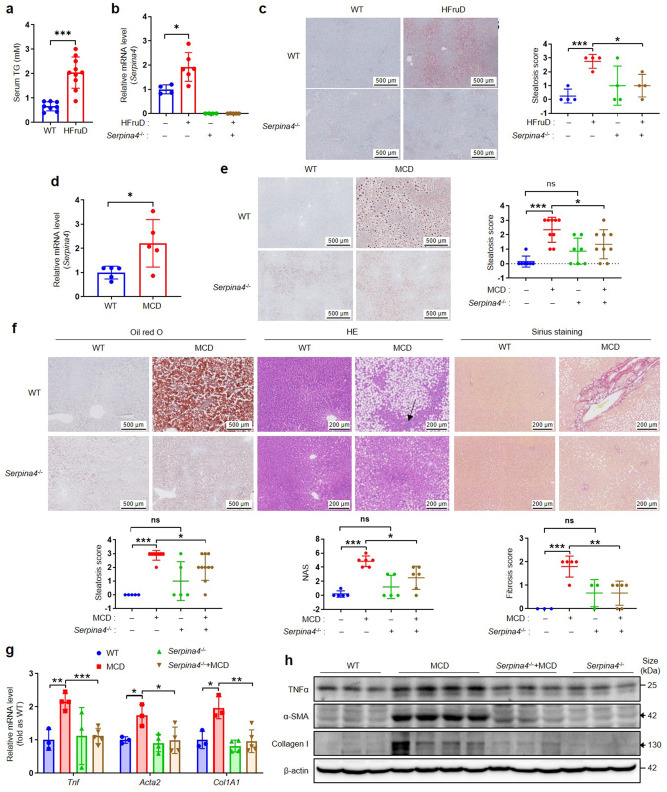
KAL knockout improves hepatic steatosis, inflammatory infiltration and fibrosis of NAFLD rats. **a** The serum TG of rats fed with HFruD for 16 weeks. **b**, **c** mRNA levels of *Serpina4*, Oil red O staining and steatosis score of livers from the rats fed with HFruD for 16 weeks. **d** mRNA levels of *Serpina4* in the livers from rats fed with MCD. **e** Oil red O staining and steatosis score of livers from the rats fed with MCD for 4 weeks. **f** Oil red O staining and steatosis score, H&E staining and NAS, Sirius staining and fibrosis scores of livers from the rats fed with MCD for 10 weeks. **g** The mRNA levels of *Tnf*, *Acta2* and *Col1a1* in the livers of rats fed with MCD for 10 weeks. **h** Immunoblot of TNFα, α-SMA, and Collagen I in the liver tissues from rats fed with MCD for 10 weeks, the black arrow represents the destination blots. Scale bar for Oil red O staining: 500 μm, for H&E staining and Sirius staining: 200 μm. Data are expressed as mean ± SD of no less than 4 rats per group. *p < 0.05, **p < 0.01, ***p < 0.001

As previously mentioned, KAL is a secreted protein. To elucidate the role of KAL in NAFLD, we generated KAL gene knockout (*Serpina4*^−/−^) rats and fed them with a HFruD for 16 weeks to induce an HTG non-obese NAFLD model (Fig. 3a), and found that the expression of KAL in the liver was higher in HFruD rats; *Serpina4*^−/−^ rats showed a significant improvement in hepatic steatosis in HFruD rats (Fig. 3b-c). Furthermore, fed *Serpina4*^−/−^ rats with a MCD diet for 4 weeks to induce hepatic steatosis and 10 weeks to NASH, respectively. We observed that the expression of KAL in the liver was also higher in MCD diet-induced NAFLD rats (Fig. 3d); *Serpina4*^−/−^ rats showed a significant improvement in hepatic steatosis, inflammation, and collagen fiber deposition in MCD-induced NAFLD rats (Fig. 3e-f). Additionally, *Serpina4*^−/−^ down-regulated the expression of TNFα, α-SMA and Collagen I in the liver tissue of MCD-induced NAFLD rats (Fig. 3g-h, supplementary Fig. 2o). These loss-of-function experiments provide evidence that high levels of KAL contribute to NAFLD.

### KAL causes hepatic steatosis by down-regulating both CGI-58 and ATGL, and inflammation mainly by CGI-58

**Fig. 4:**
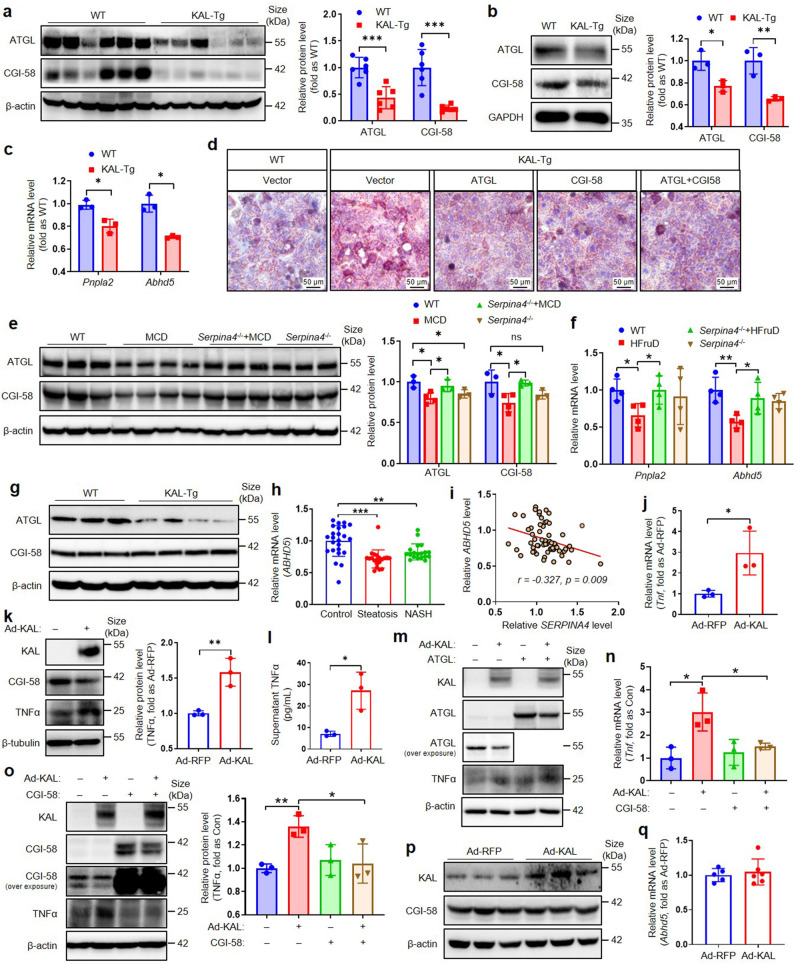
KAL causes hepatic steatosis by down-regulating both CGI-58 and ATGL, and inflammation mainly by CGI-58. **a** Representative immunoblot and quantification of ATGL and CGI-58 in liver tissues from 6-month-old mice. **b**, **c** Representative immunoblot and quantification of protein levels, mRNA levels of ATGL and CGI-58 in primary hepatocytes cultured from WT or KAL-Tg mice. **d** Oil red O staining of primary hepatocytes cultured from WT and KAL-Tg mice and transfected with ATGL and/or CGI-58 plasmids for 48 h. Scale bar: 50 μm. **e**, **f** The levels of ATGL and CGI-58 in liver tissues from rats fed with MCD for 10 weeks (e) or HFruD for 16 weeks(f). **g** Representative immunoblot of ATGL and CGI-58 in liver tissues from 3-month-old mice. **h** mRNA levels of *ABHD5* in liver tissue from patients with hepatic steatosis or NASH (Control, n = 24; steatosis, n = 20; NASH, n = 19 for GEO accession number GSE89632). **i** Correlation analysis of mRNA levels of *ABHD5* and *SERPINA4*. **j**–**l** Representative immunoblot and quantification of TNFα, mRNA levels of TNFα, and supernatant TNFα levels in primary hepatocytes treated with Ad-KAL or Ad-RFP for 48 h. **m**–**o** Representative immunoblot and quantification of TNFα, mRNA levels of TNFα in primary hepatocytes transfected with Ad-KAL and ATGL or CGI-58 plasmid for 48 h. **p**, **q** Protein levels and mRNA levels of CGI-58 in Raw macrophagocytes. Data represent the mean ± SD of three independent experiments. *p < 0.05, **p < 0.01, ***p < 0.001

Our RNAseq result of primary hepatocytes from 6-month-old WT and KAL-Tg mice indicated the downregulation of ATGL (encoded by the *Pnpla2*) and CGI-58 (encoded by the *Abhd5*) (supplementary Fig. 3a-b) (GSA dataset CRA014927), which are closed related with NAFLD. Hepatic ectopic deposition of lipid droplet-like triglycerides is influenced by several factors, including increased uptake and de novo synthesis of FFA, reduced β-oxidation of FFA, reduced TG hydrolysis, and extracellular transport through VLDL.^2^ Our study revealed that KAL did not increase FFA synthesis and uptake, had no effect on the FFA β-oxidation and the critical molecules of VLDL synthesis (supplementary Fig. 3c-d), nor did it alter the Akt signaling pathway or hepatic glucose transporter (supplementary Fig. 3e). Consistent with RNAseq results, the expressions of ATGL, a critical enzyme for TG hydrolysis, and its co-activator CGI-58 were significantly decreased by KAL (Fig. 4a). Moreover, ATGL and CGI-58 were down-regulated in primary hepatocytes treated with Ad-KAL or primary hepatocytes from KAL-Tg mice (Fig. 4b-c, supplementary Fig. 3f-g). Overexpression of ATGL and/or CGI-58 could reduce the lipid droplet induced by KAL in hepatocytes (Fig. 4d). Similarly, decreased ATGL and CGI-58 expression in liver tissue was observed in MCD and HFruD-induced NAFLD rats, which was effectively rescued by *Serpina4*^−/−^ (Fig. 4e-f). Therefore, it is suggested that KAL inhibited hepatic TG hydrolysis by suppressing CGI-58 and ATGL, leading to hepatic steatosis.

Further, we observed that the expression of ATGL, but not CGI-58, had begun to decline in the liver tissues of 3-month-old KAL-Tg mice (Fig. 4g, supplementary Fig. 3h). In addition, we analyzed a human NAFLD data set (GSE89632) and found that CGI-58 expression was decreased in liver tissues of patients with hepatic steatosis and NASH and was significantly negatively correlated with KAL (Fig. 4h-i). Studies have shown that deficiency of liver ATGL causes hepatic steatosis but not NASH or fibrosis,^17^ whereas hepatic CGI-58 deficient mice exhibit pronounced hepatic steatosis and later develop NASH and liver fibrosis, accompanied by increases in TNFα and α-SMA.^18^ Individuals with CGI-58 loss-of-function mutations typically experience severe hepatic steatosis, NASH, and cirrhosis.^19^ Our results showed that KAL up-regulated the expression and secretion of TNFα in primary hepatocytes (Fig. 4j-l), which could be inhibited by CGI-58 but not ATGL (Fig. 4m–o). Furthermore, KAL did not affect the expression of CGI-58 in macrophages (Fig. 4p-q). Thus, our findings suggest that KAL induces the expression of TNFα by downregulating CGI-58 in hepatocytes and leading to inflammation through an ATGL-independent mechanism.

### KAL induces nuclear translocation of NF-κB p65 by reducing its binding to CGI-58 in hepatocytes

In relation to the upregulation of TNFα expression resulting from the reduction of CGI-58, a question was raised regarding the mechanism involved. Our investigation first focused on NF-κB, a crucial inflammatory signaling pathway implicated in chronic inflammation associated with NAFLD^11^ and a known inducer of TNFα transcription.^20^ Previous research has indicated that the activation of the NF-κB signaling pathway by KAL results in inflammation in diabetic wound tissues.^13^ However, whether reducing CGI-58 would activate the NF-κB signaling pathway and lead to the upregulation of TNFα induced by KAL is unknown.

**Fig. 5:**
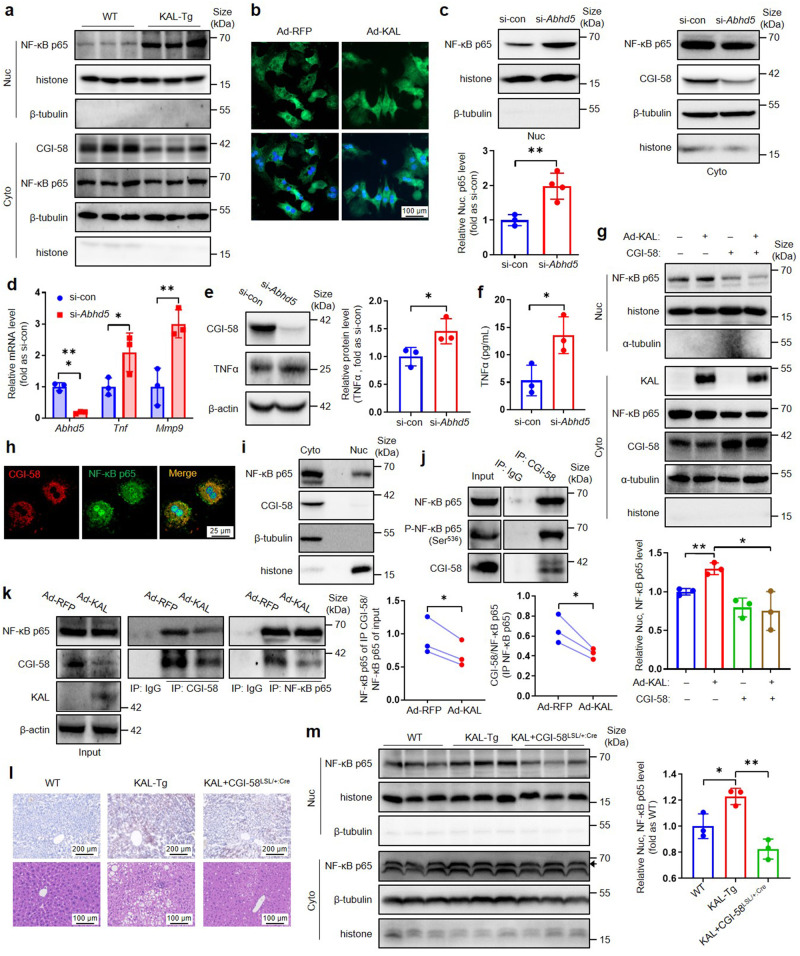
KAL induces nuclear translocation of NF-κB p65 by reducing its binding to CGI-58 in hepatocytes. **a** NF-κB p65 levels in cytosolic (Cyto.) and nuclear (Nuc.) extracts in liver tissues from 10-month-old mice. **b** NF-κB p65 (green) immunostaining in primary hepatocytes treated with Ad-KAL for 48 h. Scale bar: 100 μm. **c–f** Representative immunoblot and quantification of NF-κB p65 in nuclear extracts (**c**), mRNA levels of TNFα (**d**), representative immunoblot and quantification of TNFα (**e**), and supernatant TNFα levels (**f**) in hepatocytes treated with si-*Abhd5* for 48 h. **g** Representative immunoblot and quantification of NF-κB p65 in nuclear extracts of primary hepatocytes transfected with KAL and CGI-58 plasmid for 48 h. **h** NF-κB p65 (green) and CGI-58 (red) immunostaining in primary hepatocytes. Images were acquired under a laser-scanning confocal microscope. Scale bar: 25 μm. **i** NF-κB p65 and CGI-58 levels in Cyto and Nuc extracts of primary hepatocytes. **j** NF-κB p65 immunoblotting after immunoprecipitation (IP) for CGI-58 in primary hepatocytes, IP for IgG as the negative control. **k** The co-IP blot and quantification of CGI-58 and NF-κB p65 in primary hepatocytes treated with Ad-KAL for 48 h, IP for IgG as the negative control. **l**, **m** Oil red O staining, H&E staining and nuclear NF-κB p65 levels in liver tissues of hepatic CGI-58-overexpressing KAL-Tg mice (KAL + CGI-58^LSL/+:Cre^), the black arrow represents the destination blots. Scale bar for Oil red O staining: 200 μm, for H&E staining: 100 μm. Data represent the mean ± SD of three independent experiments. *p < 0.05, **p < 0.01

It is essential for NF-κB p65 to translocate into the nucleus to induce gene expression.^21^ Our findings indicate a significant increase in the nuclear translocation of NF-κB p65 in liver tissues of KAL-Tg mice and primary hepatocytes treated with Ad-KAL (Fig. 5a-b, supplementary Fig. 4a-c). Knocking down CGI-58 led to a notable increase in the nuclear translocation of NF-κB p65, along with the expression of TNFα and matrix metalloproteinase (MMP9), a well-established target of NF-κB, in primary hepatocytes (Fig. 5c-f). Furthermore, overexpressing CGI-58 could block the nuclear translocation of NF-κB p65 induced by KAL (Fig. 5g). Taken together, our results demonstrate that reducing CGI-58 leads to the activation of the NF-κB/TNFα signaling pathway.

To further explore how CGI-58 regulates the nuclear translocation of NF-κB p65, we performed immunofluorescence staining with NF-κB p65 and CGI-58 and found their co-localization in the cytoplasm; CGI-58 was only localized in the cytoplasm (Fig. 5h, i, supplementary Fig. 4d). Immunoprecipitation (IP) for CGI-58 in hepatocyte cytoplasm fractions identified an interaction between NF-κB p65 and p-NF-κB p65 with CGI-58, which was impaired by Ad-KAL or si-*Abhd5* (Fig. 5j, k, supplementary Fig. 4e). We also performed co-IP to identify the NF-κB subunits that mediate NF-κB activation in the absence of CGI-58 and found that the p65-p50 interactions were potentiated by reduced CGI-58 in hepatocytes (Supplementary Fig. 4f). Thus, our findings suggest that KAL down-regulates CGI-58, which interacts with NF-κB p65 and sequesters p65 in the cytoplasm, subsequently releasing p65 to facilitate its nuclear translocation.

To further investigate the key role of CGI-58 in hepatocytes regarding hepatic steatosis and inflammation induced by KAL, we constructed hepatic-specific CGI-58-transgenic mice (CGI-58^LSL/+:Cre^), which were bred with KAL-Tg mice to construct the hepatic CGI-58-overexpressing KAL-Tg mouse model (KAL + CGI-58^LSL/+:Cre^). Our results indicate that hepatic CGI-58 overexpression significantly reversed hepatic steatosis, inflammation, and the content of nuclear NF-κB p65 in KAL-Tg mice (Fig. 5l, m). It suggests that KAL induces NASH primarily through CGI-58.

### KAL down-regulates ATGL and CGI-58 by inhibiting PPARγ or inducing KLF4, respectively

**Fig. 6:**
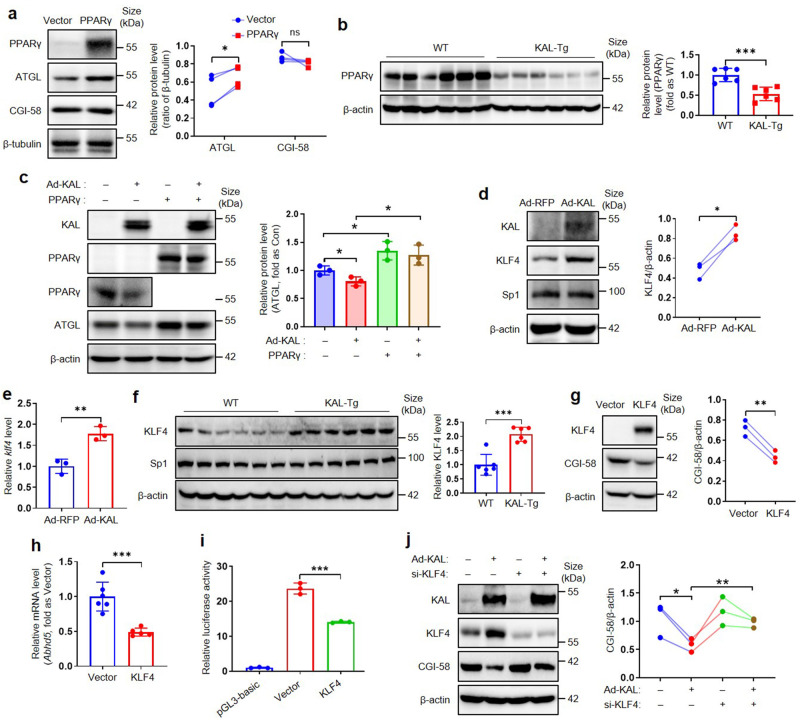
KAL down-regulates ATGL and CGI-58 by inhibiting PPARγ and up-regulating KLF4, respectively. **a** PPARγ, ATGL and CGI-58 levels in primary hepatocytes transfected with PPARγ plasmid for 48 h. **b** PPARγ levels in liver tissues of 6-month-old mice. **c** Representative immunoblot and quantification of ATGL in primary hepatocytes transfected with Ad-KAL and PPARγ plasmid for 48 h. **d**, **e** Representative immunoblot and quantification of protein levels, and mRNA levels of KLF4 in primary hepatocytes transfected with Ad-KAL for 48 h. **f** Representative immunoblot and quantification of KLF4 and Sp1 in liver tissues of 16-month-old mice. **g**, **h** Representative immunoblot and quantification of CGI-58, mRNA levels of CGI-58 in primary hepatocytes transfected with KLF4 plasmid for 24 h. **i** Luciferase reporter assays of primary hepatocytes transfected with CGI-58 promoter reporters and KLF4 plasmids for 24 h. **j** Representative immunoblot and quantification of CGI-58 in primary hepatocytes treated with Ad-KAL and si-KLF4 for 48 h. Data represent the mean ± SD of three independent experiments. *p < 0.05, **p < 0.01, ***p < 0.001

The underlying mechanism by which KAL down-regulated ATGL and CGI-58 requires further clarification. As shown in Fig. 4c and supplementary Fig. 3g, KAL inhibited the transcription of ATGL and CGI-58 in hepatocytes. It was reported that PPARγ could directly bind to the promoter of ATGL and enhance its transcription;^22^ Bioinformatics prediction indicated that PPARγ may also bind to the CGI-58 promoter (supplementary Fig. 5a); Moreover, PPARγ is closely related to NAFLD.^23^ However, in hepatocytes, PPARγ promoted the expression of ATGL but not CGI-58 (Fig. 6a). KAL suppressed the expression of PPARγ in liver tissues, and *Serpina4*^*-/-*^ significantly improved the downregulation of PPARγ in MCD-induced NAFLD rats (Fig. 6b, supplementary Fig. 5b). Overexpression of PPARγ could block the downregulation of ATGL induced by KAL in hepatocytes (Fig. 6c). Therefore, it is suggested that KAL down-regulated ATGL by inhibiting PPARγ.

Furthermore, we found that the CGI-58 promotor contains a G/C-rich element. The classical transcription factors that could bind to this element were KLF4 and Sp1.^24^ KAL was found to up-regulate KLF4 but not Sp1 in hepatocytes (Fig. 6d-e); KLF4, not Sp1, was significantly elevated in liver tissues of KAL-Tg mice (Fig. 6f). Furthermore, KLF4 inhibited the expression of CGI-58 and the activity of its promoter in hepatocytes (Fig. 6g-i). Silencing of KLF4 reversed the downregulation of CGI-58 induced by KAL in hepatocytes (Fig. 6j). Hence, it is suggested that KAL down-regulated CGI-58 by inducing KLF4.

### KAL regulates KLF4 and PPARγ by LRP6/Gαs/PKA/GSK3β signals

**Fig. 7:**
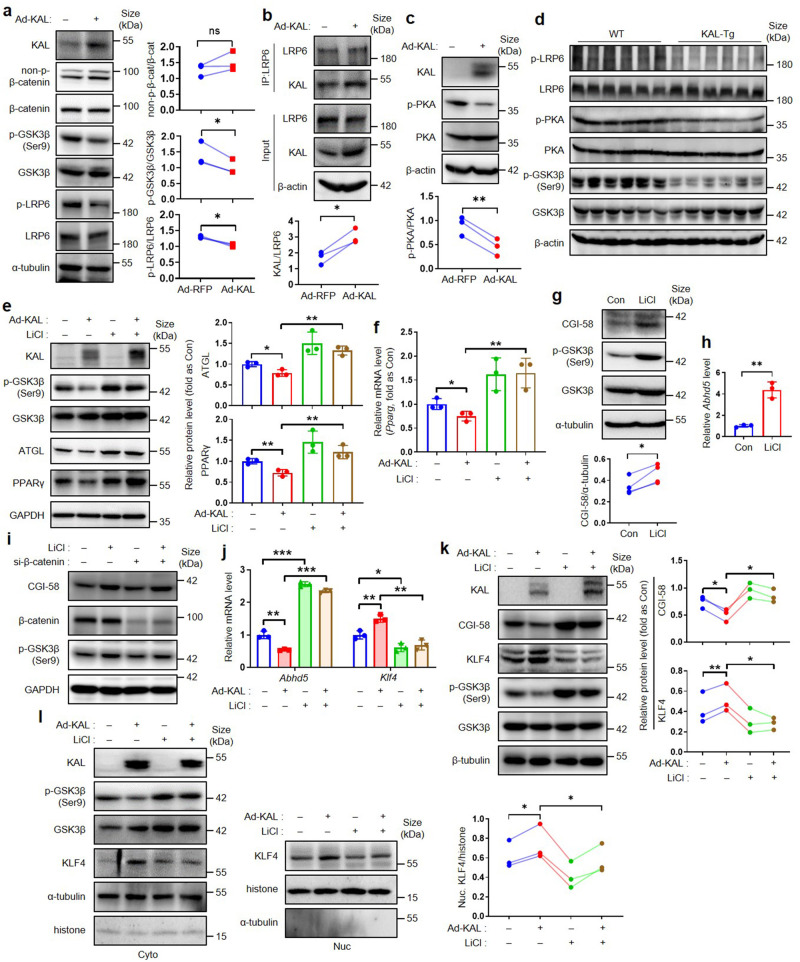
KAL regulates KLF4 and PPARγ by activating GSK3β. **a–c** Protein levels of non-p-β-catenin, p-GSK3β, p-LRP6 (**a**), p-PKA (**c**), and the IP blot of KAL and LRP6 (**b**) in hepatocytes treated with Ad-KAL for 48 h. **d** Protein levels of p-LRP6, p-PKA, p-GSK3β in liver tissues of 16-month-old mice. **e**, **f** Representative immunoblot and quantification of protein levels, mRNA levels of ATGL and PPARγ in hepatocytes treated with Ad-KAL and LiCl (20 mM) for 48 h. **g**, **h** Protein levels and mRNA levels of CGI-58 in hepatocytes treated with LiCl (20 mM) for 48 h. **i** Protein level of CGI-58 in hepatocytes treated with LiCl (20 mM) and si-β-catenin for 48 h. **j**, **k** Representative immunoblot and quantification of protein levels, mRNA levels of CGI-58 and KLF4 in hepatocytes treated with Ad-KAL and LiCl (20 mM) for 48 h. **l** Representative immunoblot and quantification of KLF4 in nuclear extracts of hepatocytes treated with Ad-KAL and LiCl (20 mM) for 48 h. Data represent the mean ± SD of three independent experiments. *p < 0.05, **p < 0.01, ***p < 0.001

KAL is a well-known suppressor of the GSK3β/β-catenin signaling pathway by binding with low-density lipoprotein receptor-related protein 6 (LRP6).^25^ Surprisingly, our findings suggest that KAL does not affect the expression of Wnt ligands, the composition of DVL/GSK3β/β-catenin, and β-catenin activity, but significantly down-regulates the phosphorylation of both LRP6 and glycogen synthase kinase-3 (GSK3β) markedly (Fig. 7a, supplementary Fig. 6a-b). Hepatocytes express various G-protein-coupled receptors (GPCRs) such as the glucagon receptor and thyrotropin-releasing hormone receptor;^26^ LRP6, a coreceptor of GPCRs, is required for the activation of protein kinase A (PKA) under the stimulation of different GPCR ligands through the membrane G protein α(s) subunit (Gαs);^27^ What is more, PKA was the classical kinases that could phosphorylate GSK3β and leading to its inactivation.^28^ Our results indicate that KAL can bind to LRP6 in hepatocytes, and this binding is enhanced upon KAL overexpression (Fig. 7b); KAL also disrupts the localization of Gαs to the plasma membrane (supplementary Fig. 6c) and inhibits the phosphorylation of PKA in hepatocytes, treatment with the PKA agonist Dibutyryl-cAMP (db-cAMP) blocks the downregulation of p-GSK3β induced by KAL (Fig. 7c, supplementary Fig. 6d); Moreover, the phosphorylation of LRP6, PKA, and GSK3β is reduced in the livers of KAL-Tg mice (Fig. 7d, supplementary Fig. 6e). Therefore, we propose that KAL may inhibit LRP6/Gαs/PKA/GSK3β signaling.

It has been reported that PPARγ is downstream of GSK3β.^29^ Inhibition of GSK3β with lithium chloride (LiCl) can block the downregulation of PPARγ and ATGL induced by KAL (Fig. 7e, f). Additionally, LiCl induces the expression of CGI-58, which is not improved by β-catenin interference (Fig. 7g–i). Furthermore, LiCl significantly disrupts the regulatory effects of KAL on CGI-58 and KLF4, as well as the nuclear translocation of KLF4 (Fig. 7j–l). Therefore, we suggest that KAL regulates KLF4 and PPARγ by activating GSK3β.

### High FFA reverses the downregulation of KAL induced by T3, and fenofibrate down-regulates KAL in hepatocytes

**Fig. 8:**
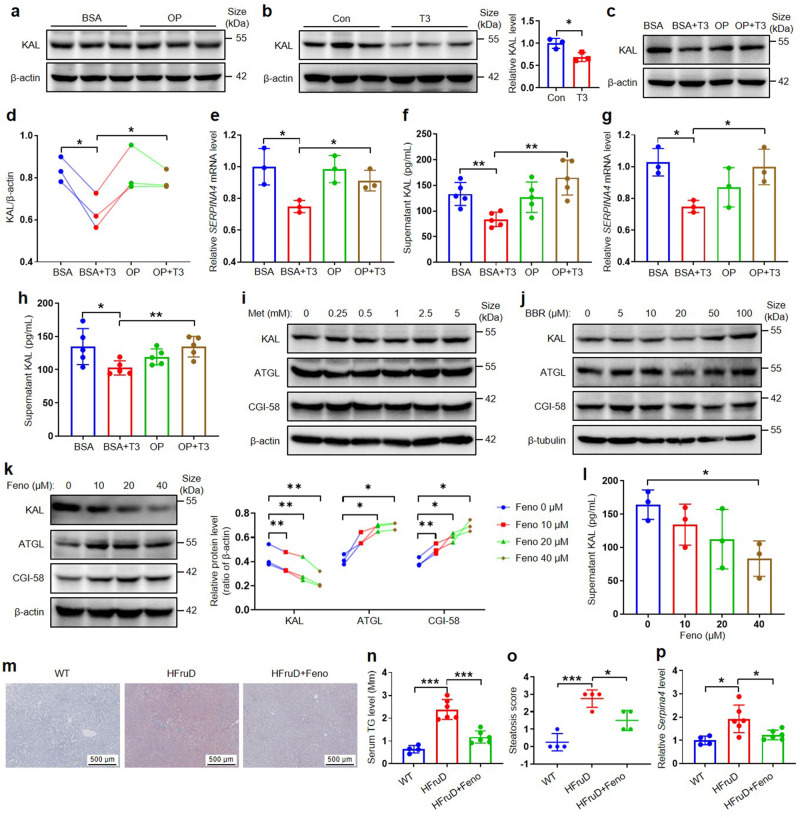
High FFA reverses the downregulation of KAL induced by T3 and Fenofibrate down-regulates KAL in hepatocytes. **a** Protein levels of KAL in L-02 cells treated with OP (250 μM Oleic acid add 250 μM Palmitic acid) or BSA (Bovine serum albumin) for 24 h. **b** Protein levels of KAL in L-02 cells treated with T3 (25 nM) for 24 h. **c**–**f** Representative immunoblot and quantification of KAL, mRNA levels of KAL, and supernatant KAL levels in L-02 cells treated with T3 (25 nM) with/without OP for 24 h. **g**–**h** mRNA levels of KAL, supernatant KAL levels in MIHA cells treated with T3 (25 nM) with/without OP for 24 h. **i**–**k** Representative immunoblot of KAL, ATGL, CGI-58 in L-02 cells treated with Metformin (Met), Berberine (BBR), or Fenofibrate (Feno). **l** Supernatant KAL levels in L-02 cells treated with Feno. **m**–**p** Oil red O staining, serum TG level, steatosis score, and the mRNA level of KAL in livers from the rats fed with HFruD for 12 weeks, followed by the treatment of 100 mg/kg Feno and HFruD for 4 weeks. Scale bar: 500 μm. Data represent the mean ± SD of three independent experiments. *p < 0.05, **p < 0.01, ***p < 0.001

The regulation of KAL gene expression remains poorly understood, leading to the question of how KAL is up-regulated. It has been reported that HTG is accompanied by higher levels of FFA, a product of lipid metabolic dysfunction,^30^ and NAFLD patients had significantly higher serum FFA levels than controls.^31^ FFA examined in our HLP subjects was positively correlated with plasma KAL levels (supplementary Fig. 1e). Our findings indicate that high levels of FFA alone do not affect KAL expression (Fig. 8a). A previous study has shown that *Serpina3c*, a member of the same family as KAL, is negatively regulated by thyroid hormone T3: T3 binds to thyroid hormone receptor (TR), then liganded TR recruits nuclear receptor corepressor and represses the expression of *Serpina3c*.^32^ Our results show that T3 also down-regulates KAL expression in hepatocytes (Fig. 8b). Additionally, T3 binding to TR is significantly inhibited when plasma and cell FFA are increased.^33^ Furthermore, our results demonstrate that high FFA can counteract the down-regulation of KAL expression and secretion induced by T3 in hepatocytes (Fig. 8c-h). Most T3 is produced from the conversion of thyroid hormone T4 in peripheral tissues, especially the liver and kidney.^34^ Thus, these results suggest that high FFA, a product of lipid metabolic dysfunction, can elevate KAL and indicate that KAL is a linker between HLP and NAFLD.

We also investigated whether compound drugs with improved hepatic steatosis and/or lipid-lowering effects, such as Metformin, Berberine, and Fenofibrate, could decrease KAL expression. Our findings show that only Fenofibrate, a kind of drug for severe HTG, but not Metformin and Berberine, can down-regulate the expression and secretion of KAL and subsequently improve the expression of ATGL and CGI-58 in hepatocytes (Fig. 8i–l). Further, intragastric administration of 100 mg/kg Fenofibrate daily reversed serum TG level, hepatic steatosis, and upregulation of KAL in the livers of HfruD rats (Fig. 8m–p). These results suggest that triglyceride-lowering drugs may benefit NAFLD by decreasing KAL.

## Discussion

The transition from hepatic steatosis to NASH plays a crucial role in the progression of NAFLD to irreversible liver damage.^8^ However, the key molecules and signaling pathways involved in this process are not yet fully understood. NAFLD is more prevalent in individuals with HTG than those with HTC (High total cholesterol);^3^ Our data showed that the proportion of subjects with elevated KAL was also higher in the HTG population than in the HTC population (Fig. 1c). Non-obese NAFLD usually has higher TG levels when compared with matched controls.^6^ Our study focused on the non-obese NAFLD, and found that KAL was significantly elevated in HTG subjects and NAFLD patients and positively correlated with TG; Additionally, feeding with HFruD is the classical animal model of HTG, and KAL deficiency could significantly reverse the hepatic steatosis of HFruD rats; FFA, the consequent product of lipid metabolic dysfunction, cooperated with T3 and up-regulated KAL in hepatocytes. Therefore, we believe that lipid metabolic dysfunction is the cause of elevated KAL in HLP and NAFLD patients and suggests that KAL may be the causative contributor that mediates HLP to NAFLD.

The NAFLD patients included in this project were mild, and hepatocyte necrosis had not yet occurred (Fig. 1), enabling us to examine the change and role of KAL in the early stages of the disease. It has been reported that KAL was lower in the serum of liver cirrhosis patients.^35^ As mentioned above, KAL is mainly produced by hepatocytes,^12^ so KAL levels are expected to decrease in the late stage of liver cirrhosis when hepatocytes are necrotic. Our results indicate that elevated KAL could initiate liver injury and induce irreversible NASH, which in turn leads to a decline of KAL.

Mechanistically, KAL down-regulated the expression of ATGL and CGI-58, which play critical roles in TG hydrolysis and hepatic steatosis.^36,37^ Furthermore, KAL inhibited the transcription of ATGL by down-regulating PPARγ. Previous clinical trials have shown that Pioglitazone, a PPARγ agonist, can improve hepatic steatosis in NAFLD patients but does not affect NASH or liver fibrosis.^38^ Our findings suggest that PPARγ may only regulate the transcription of ATGL but not CGI-58, which may explain why Pioglitazone is ineffective in treating NASH and liver fibrosis.

Previous studies on CGI-58 transcription regulation have mainly focused on Qinchuan cattle.^39,40^ Our study identified KLF4 as a transcription suppressor of CGI-58 and demonstrated that KAL down-regulates CGI-58 by elevating KLF4 for the first time. The importance of CGI-58 in hepatic steatosis, NASH, and fibrosis has been clarified through studies on CGI-58 knockout mice and the CGI-58 mutation population.^18,41^ Furthermore, the deficiency of liver ATGL caused hepatic steatosis, but not NASH or fibrosis,^17,42^ suggesting that CGI-58 could regulate hepatic inflammation and fibrosis through an ATGL-independent mechanism.^43^ However, the mechanism of CGI-58 in regulating NASH and fibrosis is still unclear. TNFα was increased in the liver of hepatocyte-specific CGI-58 knockout mice,^18,44^ similar to KAL-Tg mice. In addition, it was reported that CGI-58 could suppress the nuclear translocation of NF-κB p65 in tumor-associated macrophages.^45^ We clarified for the first time that CGI-58 could bind NF-κB p65 and sequester it in the cytoplasm. Diminishing CGI-58 induced by KAL released p65 to facilitate its nuclear translocation and subsequently induced the expression of TNFα. At the same time, it is reported that phosphorylation of NF-κB p65 can enhance its transcriptional activity and promote its nuclear translocation,^46^ and our results showed that CGI-58 could also bind to p-NF-κB p65 in the cytoplasm (Fig. 5j). Therefore, we speculate that even if the NF-κB p65 was phosphorylated, CGI-58 could still sequester it in the cytoplasm, which indicates that CGI-58 is a novel endogenous inhibitor of the NF-κB signaling pathway in hepatocytes.

Although Kupffer cells (macrophages) are known to promote an inflammatory response in the development of NASH, it has also been reported that injured hepatocytes produce pro-inflammatory cytokines and chemokines.^47^ Since hepatocyte-specific CGI-58 knockout mice exhibited inflammation and fibrosis in the liver, our experiment shows that KAL specifically down-regulates CGI-58 and induces TNFα in hepatocytes but not macrophages, and hepatocyte-specific CGI-58-overexpression could improve the hepatic steatosis and inflammation of KAL-Tg mice. Thus, KAL primarily targets hepatocytes and plays a direct pro-inflammatory role.

Previous studies have reported that KAL can inhibit inflammatory responses in hypertensive rats, mice with multifactorial sepsis, and endotoxemia,^48–50^ suggesting that KAL has anti-inflammatory effects that contradict our findings. These studies were conducted in acute inflammation models induced by lipopolysaccharides (LPS), whereas NASH is a chronic inflammatory condition. Moreover, KAL has a dual role in angiogenesis, apoptosis, oxidative stress, and inflammation.^51^ At physiological concentrations, KAL is not harmful, but either too low or too high concentrations can be damaging. For instance, elevated KAL levels have been found in patients with type I and type II diabetes, and our recent study showed that elevated KAL can activate the NF-κB signaling pathway to cause chronic inflammation in diabetic wound tissues.^13,14^ Similarly, our present study shows that elevated KAL leads to NASH by down-regulating CGI58, reflecting a pro-inflammatory effect. The study adds to our understanding of the dual role of KAL in physiological and pathological conditions.

Our study also investigated the potential therapeutic effects of drugs on KAL levels in NAFLD. Metformin and Berberine have demonstrated a broad spectrum of pharmacological effects and potential therapeutic effects on NAFLD; Fenofibrate is a kind of drug for treating HLP, especially severe HTG, and can reduce the content of FFA while reducing TG.^52^ We found that Fenofibrate, but not Metformin and Berberine, down-regulates KAL levels. Metformin treatment is effective for diabetic NAFLD patients;^53^ However, a meta-analysis showed that metformin was not associated with liver histologic improvement in NASH patients without diabetes.^54^ Berberine improves hepatic steatosis but has no effect on NASH;^55^ moreover, it is reported that LDL-lowering drugs Statins do not respond well to NASH.^38^ Previous clinical trials of Fenofibrate on NAFLD have yielded mixed results, with some studies reporting improvements in TG, AST, ALT, hepatic steatosis, inflammation and liver stiffness, while others found no significant benefits.^56–60^ Our findings suggest that drugs with TG-lowering or FFA-lowering effects may effectively treat NAFLD/NASH by down-regulating KAL levels. Therefore, Fenofibrate may be a promising treatment option for NAFLD patients with elevated KAL levels, yet clinical trials are needed to confirm its efficacy.

**Fig. 9:**
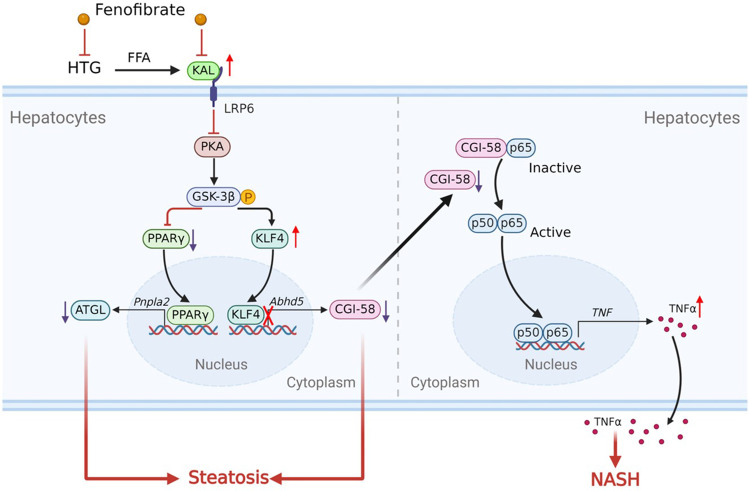
A schematic model of the contributor and potential mechanism of KAL in the occurrence and development of NAFLD. KAL is increased in HTG subjects and NAFLD patients. FFA, the consequent product of HTG, up-regulates KAL in hepatocytes. Elevated KAL induced hepatic steatosis and NASH by down-regulating ATGL and CGI-58 by LRP6/PKA/GSK3β pathway. Consequently, diminishing CGI-58 activated NF-κB/TNFα signaling pathway. Fenofibrate may benefit NAFLD by decreasing KAL

In summary, our study suggests that elevated KAL levels contribute to the occurrence and development of NAFLD by targeting the CGI-58/NF-κB/TNFα pathway (Fig. 9). This finding enhances our understanding of the pathophysiology of NAFLD and highlights KAL as a potential therapeutic target for NAFLD treatment. Thus, targeting KAL in the early stages of NAFLD may represent a promising therapeutic strategy.

## Materials and methods

### Ethics statements

This study collected blood samples from human subjects. All participants provided written informed consent. This study was approved by the Medical Ethics Committees of Zhongshan Medical School, Sun Yat-Sen University (Approval number ZZSOM-MEC-2022-001).

The animal experimental process has been reviewed and approved by the Institutional Animal Care and Use Committee of Sun Yat-Sen University (SYSU-IACUC-2022-B0016).

### Experimental design of human subjects

We enrolled 221 healthy control and 253 HLP subjects in the Clinical Laboratory of Guangzhou First People’s Hospital and enrolled 115 participants in the Physical Examination Centre of the Third Affiliated Hospital of Sun Yat-Sen University, which included 53 NAFLD patients and 62 age-matched healthy control. According to the color ultrasound, the diagnosis of NAFLD was that more than 5% of hepatic cells appeared steatosis was considered to be NAFLD. Exclusion criteria included obesity, viral hepatitis, drug-induced liver disease, total parenteral nutrition, hepatolenticular degeneration or other liver diseases, and alcoholic fatty liver disease (daily alcohol consumption of more than 30 g for men and 20 g for women).

### Experimental design of animals

KAL knockout (*Serpina4*^−/−^) Sprague-Dawley rat strain was constructed by Guangzhou Saiye Biotechnology Co., Ltd., using CRISPR/Cas9 technology; Five exons have been identified in the rat *Serpina4* gene, with the ATG start codon in exon 2 and TAG stop codon in exon 5, and Exon 2 to exon 3 was selected as target sites (supplementary Fig. 7a). The KAL transgenic (KAL-Tg) C57BL/6 mouse strain was provided as a gift from Dr. Jianxing Ma (University of Oklahoma Health Sciences Center).^61^ KAL-Tg mice and litter wild-type mice were raised at different ages to confirm the change in the liver. In some experiments, the 6-week-old KAL-Tg mice and litter wild-type mice were fed a 60% fat calories HFD (Research Diets) for 28 weeks; and 8-week-old *Serpina4*^−/−^ rats and age-matched wild-type rats fed with 70% HFruD (ReadyDietech) for 16 weeks, MCD (Dyets) for four weeks or ten weeks. The serum contents of AST, ALT, TG, TC, LDL-C, and HDL-C were analyzed by the Hitachi Biochemistry Analyzer. In addition, the TG and fatty acid content of liver tissues were quantified by commercial kits purchased from BioAssay Systems (ETGA-200 and EFFA-100, USA).

CGI-58-transgenic mice were constructed by Shanghai Model Organisms, using CRISPR/Cas9 technology; The CAG-LSL-ABHD5-WPRE-polyA expression frame was inserted at the Rosa26 gene locus through homologous recombination (supplementary Fig. 7b-c). Then breeding them with Alb-cre mice to obtain liver-specific overexpression CGI-58 mice, which were bred with KAL-Tg mice to construct the hepatic CGI-58-overexpressing KAL-Tg mouse model (KAL + CGI-58^LSL/+:Cre^) for experiments.

All animals were fed in the Specific-pathogen-free (SPF) environment of the Experimental Animal Centre of Zhongshan School of Medicine, Sun Yat-Sen University. The use and handling of all animals strictly follow Sun Yat-Sen University’s experimental animal use guidelines.

### Cell-culture experiments

Primary hepatocytes were isolated and purified from the liver tissue of mice. Briefly, insert the catheter into the portal vein, perfuse the liver with D-Hank’s buffer (PYG0075, Boster, China) to flush the liver of red blood cells, and cut the inferior vena cave to release pressure in the liver. Then, perfuse the liver with digestion buffer (0.4 mg/mL collagenase IV, C5138, Sigma, USA); the digested livers were removed and dispersed in DMED medium with 10% fetal bovine serum. The cells were cultured in Willian’s E medium (Gibco) with 10% FBS, 2% hepatocyte maintenance culture supplement (Gibco), and 1% streptomycin/ penicillin. Glycogen Periodic Acid Schiff (PAS) staining (BP-DL036, SenBeiJia Biological Technology, Nanjing, China) and immunofluorescence of CK-18 (AF1285, Beyotime) were used to identify mouse primary hepatocytes (supplementary Fig. 8).

### Histological analyses

Frozen sections of the liver were stained with Oil red O to determine the degree of steatosis. Paraffin-embedded tissue sections were stained with Hematoxylin-eosin (HE) to determine the tissue morphology and inflammation and stained with Sirius red to determine the fibrosis. All images were captured using an automatic digital slide scanning system (AxioScan.Z1, Zeiss, DE). NAS was scored using the NASH Clinical Research Network Histologic Scoring System. NAS system and liver fibrosis score were used for semi-quantitative analysis of the main histopathological characteristics of NAFLD.^62,63^ The NAS was an unweighted scoring system based on levels of steatosis(0–3), lobular inflammation(0–3), and hepatocyte ballooning(0–2). The criteria to diagnose NASH is a NAS score of 5 or greater. The evaluation criteria are detailed in supplementary Table 3.

### Enzyme-linked immuno sorbent assay (ELISA) specific for KAL

The concentration of plasma and serum KAL were quantified by commercial ELISA kits purchased from R&D Systems (DY1669).

### Immunoprecipitation, isolation of nuclear fractions and western blot

Immunoprecipitation was performed to detect protein complex formation. Wash the cells with cold PBS and lyse with RIPA buffer (P0013D, Beyotime, China). The lysate was incubated with a primary antibody overnight. Then, Protein A/G PLUS Agarose (sc-2003, Santa Cruz) was added and further rotated for 6 h. Finally, the Agarose-antibodies-protein complex was washed with RIPA buffer three times and analyzed by western blot. Nuclear fraction was performed by Nuclear and Cytoplasmic Protein Extraction Kit (P0027, Beyotime, China) and analyzed by western blot. Antibody information is indicated in Supplementary Table 4. The results were quantified by the National Institutes of Health [NIH] ImageJ 1.34.

### Plasmids, siRNA, and transfection

Adenovirus overexpressing KAL (Ad-KAL) and adenovirus overexpressing red fluorescent protein (Ad-RFP) were purchased from Obio Technology Corp Ltd (Shanghai, China). Plasmids encoding PPARγ, ATGL, CGI-58, and KLF4 were purchased from MiaoLing Plasmid Sharing Platform (Wuhan, China). Transfection of plasmids was performed at 70%-80% confluency using Lipofectamine 3000 (L3000015, Gibco, USA) according to the manufacturer’s instructions. CGI-58 siRNA and KLF4 siRNA were purchased from RiboBio (Guangzhou, China); the Interference sequence is listed in supplementary Table 5. Transfection of siRNA was performed using Lipofectamine RNAiMAX (13778150, Gibco, USA).

### RNA isolation and real-time quantitative polymerase chain reaction (RT-qPCR)

The isolation and reverse transcription of RNA were performed by commercial kits purchased from EZBioscience (EZB-RN001-plus for tissues, B0003C for cells). RT-qPCR was performed with SYBR® Green I (Takara, China) by a Real-time fluorescent quantitative PCR system (Bio-rad, USA). The PCR primer sequences are listed in supplementary Table 5.

### Luciferase reporter assay

Luciferase assay was performed using the Dual-Luciferase Reporter Assay Kit (E1910, Promega, USA). We defined the CGI-58 promoter region spanning nucleotides −1000 to +224 as a full-length promoter, which was cloned into the KpnI/HindIII sites of pGL3-basic luciferase reporter plasmid to generate CGI-58 luciferase reporters.

### Statistical analysis

Each experiment was replicated with three or more separate samples. SPSS 21.0 was used for statistical analysis, and GraphPad Prism 7 was used for graphing. Continuous quantitative data conforming to the normal distribution were revealed as mean ± standard deviation (mean ± SD), the difference between the two groups was made by Student’s *t* test or paired *t* test, and *p* < 0.05 was considered statistically significant. The values for *N* and the specific statistical test performed for each experiment were included in the figure legends.

### Supplementary information

supplementary figures and tables

Unmodified gels

## Data Availability

The data that support the findings of this study are available from the corresponding author upon reasonable request.
