# Supplementary material for: Elevated Kallistatin promotes the occurrence and progression of non-alcoholic fatty liver disease
Source: Signal Transduct Target Ther. 2024 Mar 12;9:66. doi: 10.1038/s41392-024-01781-9 (PMC10933339; doi:10.1038/s41392-024-01781-9)
Supplement: Supplementary file 1 — supplementary figures and tables [file 41392_2024_1781_MOESM1_ESM.docx]

Supplementary Materials for

Elevated Kallistatin promotes the occurrence and progression of non-alcoholic fatty liver disease

Zhenzhen Fang^1^*, Gang Shen^1^*, Yina Wang^2^*, Fuyan Hong^1^*, Xiumei Tang^3^, Yongcheng Zeng^1^, Ting Zhang^4^, Huanyi Liu^5^, Yanmei Li^1^, Jinhong Wang^1^, Jing Zhang^1^, Anton Gao^6^, Weiwei Qi^1^, Xia Yang^1,7#^, Ti Zhou^1,8#^, Guoquan Gao^1,9#^

Correspondence to: [gaogq@mail.sysu.edu.cn](mailto:xxxxx@xxxx.xxx)

**This PDF file includes:**

Figures. S1 to S8

Tables S1 to S5


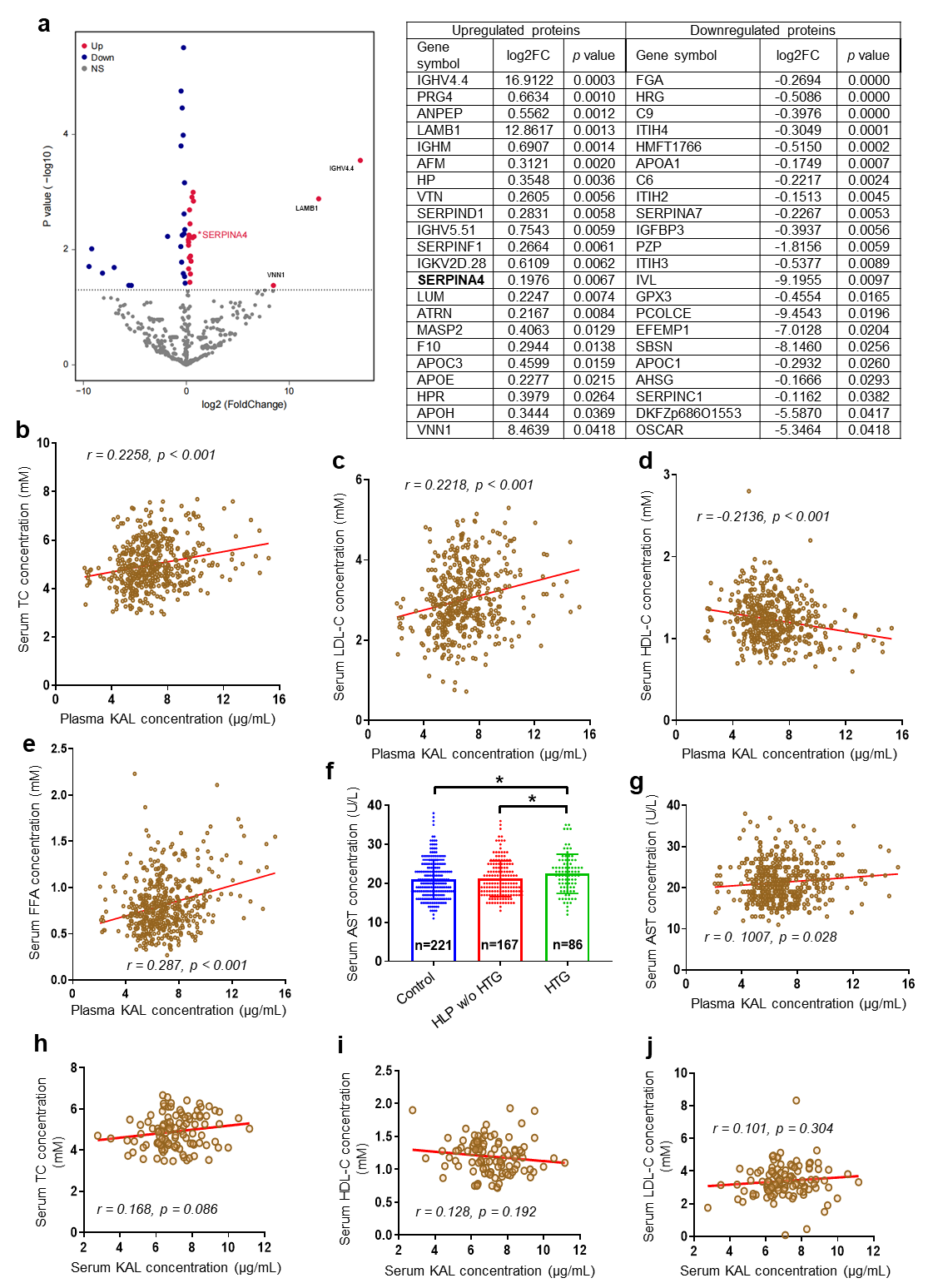


Figure. S1.

**The relationship between KAL and lipid level.** (a), Volcano plot and list showing differentially expressed proteins in the HTG group (n = 5) and Control group (n = 6). (**b-e**), Correlation analysis of plasma KAL levels and TC (b), LDL-C (c), HDL-C (d) and FFA (e) concentrations in HLP and control subjects. (**f-g**), The serum AST concentration and correlation analysis of plasma KAL and AST in control and HLP subjects. (**h-j**), Correlation analysis of serum KAL levels and TC (h), HDL-C (i) and LDL-C (j) concentrations in NAFLD and control subjects. Data are expressed as mean ± SD. Statistical analysis was performed using Student t-test.


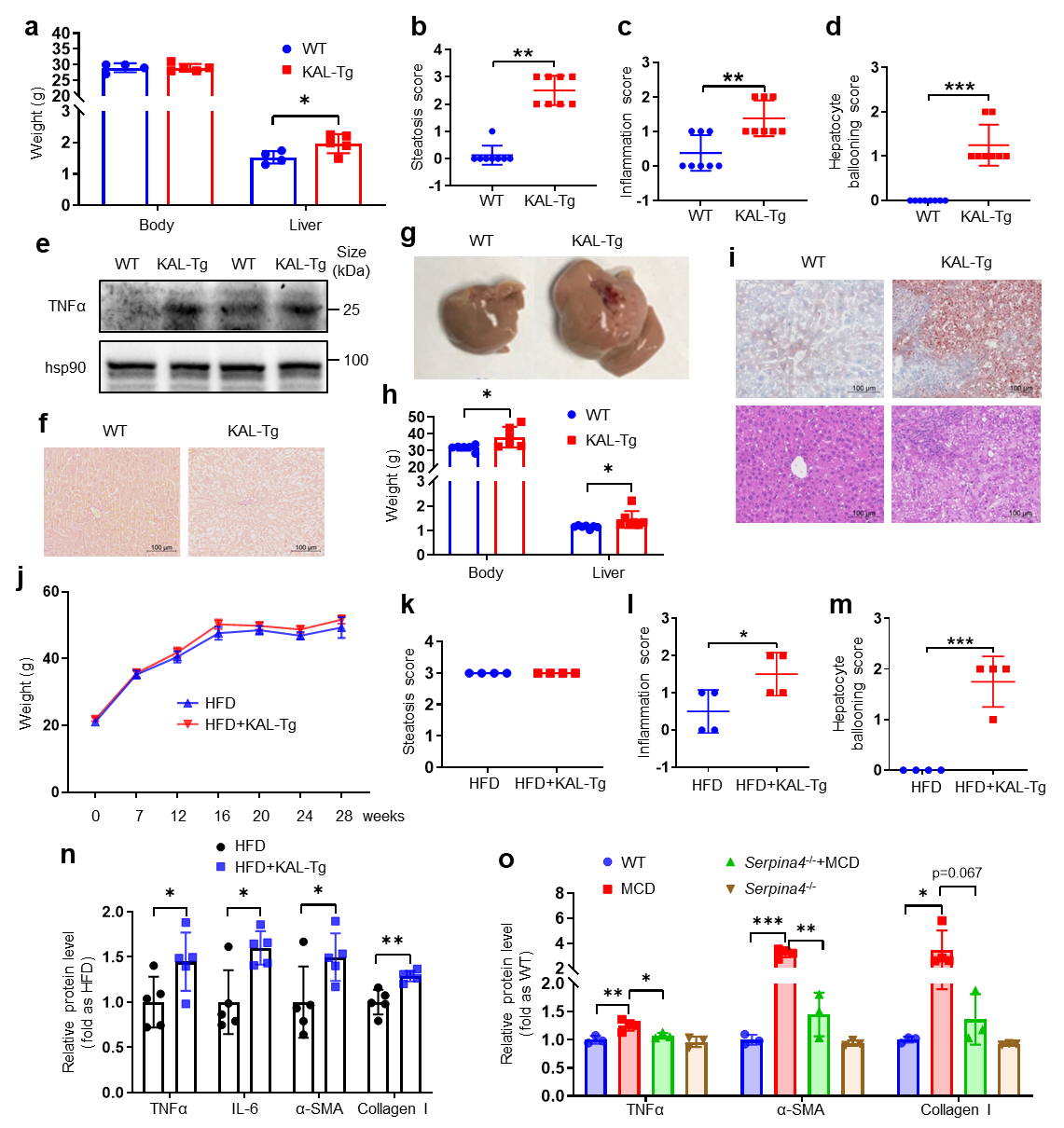


Figure. S2.

**KAL induces hepatic steatosis and NASH in chow-diet mice and progresses hepatic steatosis to NASH in HFD mice.** (**a-f**), Weight (a), steatosis scores (b), inflammation score (c), hepatocytes ballooning score (d), Representative TNFα blot images (e), Sirius staining (f) of livers from 10-month-old mice (n=8 per group). (**g-i**), Representative images (g), weight (h), Oil red O staining and H&E staining (i) of livers from 16-month-old mice (WT, n=6; KAL-g, n=5). (**j**), Weight of mice fed an HFD for 28 weeks. (**k-m**), Steatosis scores (k), inflammation score (l), hepatocytes ballooning score (m) of livers from mice fed an HFD for 28 weeks (n=4 per group). (**n**) Quantification of immunoblot in Fig.2r. (**o**) Quantification of immunoblot in Fig.3h. Data are expressed as mean ± SD. **p<0.05*, ***p<0.01*, ****p<0.001*.


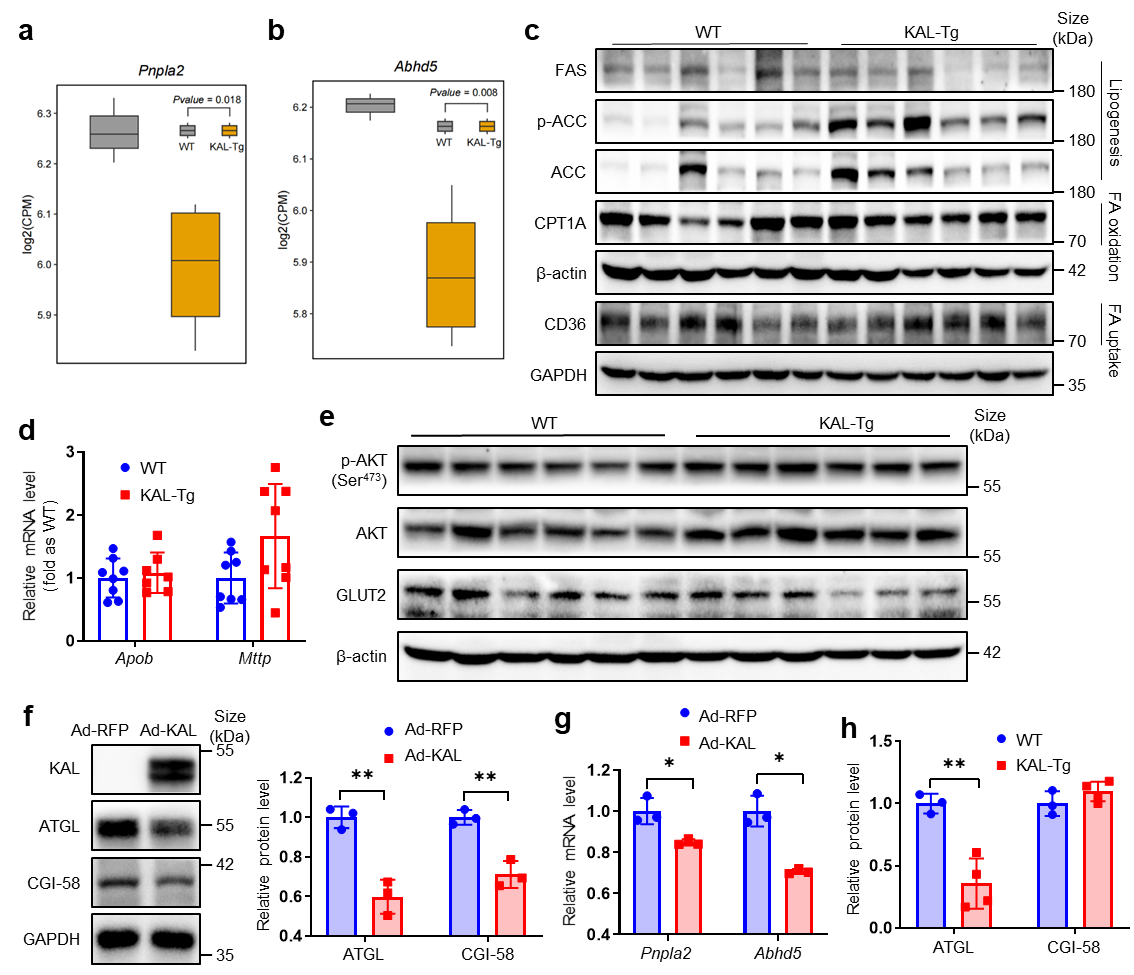


Figure. S3.

**The effect of KAL on the synthesis, β-oxidation, and uptake of FA.** (**a-b**), Boxplot of the relative mRNA levels of *Pnpla2* and *Abhd5* in primary hepatocytes from 6-month-old WT and KAL-Tg mice. (**c**), Representative immunoblot of FAS, p-ACC, ACC, CPT1A and CD36 in liver tissues from 6-month-old mice. (**d**), mRNA levels of ApoB and MTTP in liver tissues from 6-month-old mice. (**e**), Representative immunoblot of p-AKT, AKT and GLUT2 in liver tissues from 6-month-old mice. (**f-g**), Representative immunoblot and quantification of protein levels, mRNA levels of ATGL and CGI-58 in primary hepatocytes treated with Ad-KAL for 48 h. (**h**), Quantification of immunoblot in Fig.4g. Data represent the mean ± SD. **p<0.05*, ***p<0.01*.


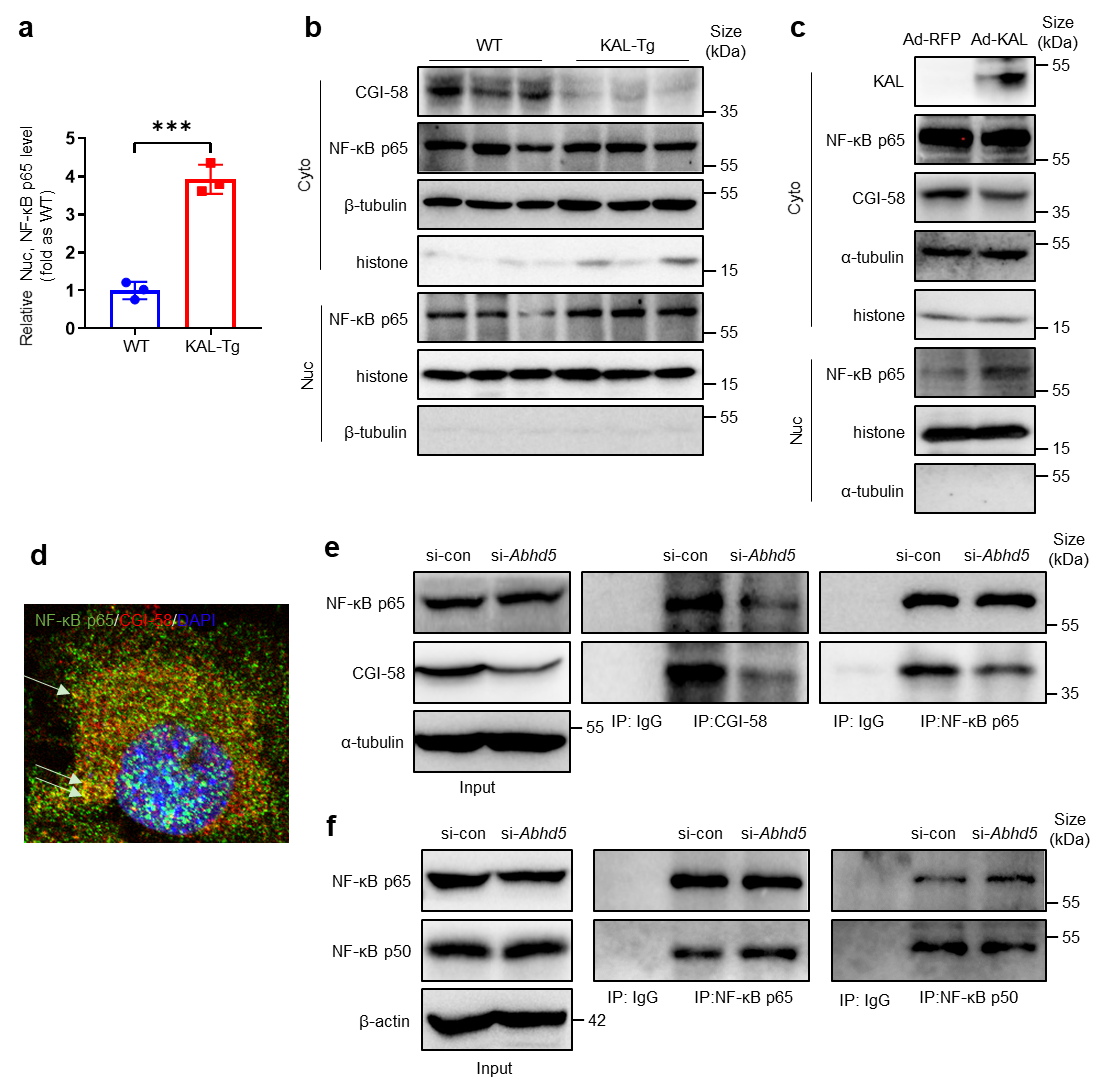


Figure. S4.

**KAL induces nuclear translocation of NF-κB p65 by reducing its binding to CGI-58 in primary hepatocytes.** (**a**), Quantification of immunoblot in Fig.5A. Data represent the mean ± SD. ***p<0.001. (**b**), Protein levels of NF-κB p65 in cytosolic (Cyto.) and nuclear (Nuc.) extracts in livers tissues from 16-month-old mice. (**c**), Protein levels of NF-κB p65 in cytosolic (Cyto.) and nuclear (Nuc.) extracts of primary hepatocytes treated with Ad-KAL for 48h. (**d**), NF-κB p65 (green) and CGI-58 (red) immunostaining in L-02 cells. Images were acquired under a laser scanning confocal microscope. (**e-f**), The co-IP blot of CGI-58 and NF-κB p65 (e), NF-κB p65 and NF-κB p50 (f) in primary hepatocytes treated with *si-Abhd5* for 48 h.


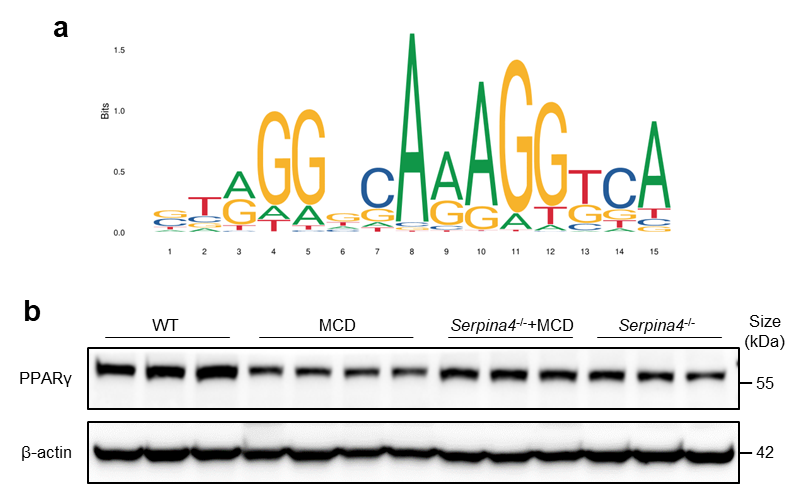


Figure. S5.

***Serpina4^-/-^* significantly improves the downregulation of PPARγ in MCD-induced NAFLD rats’ liver tissues.** (**a**), The predicted PPARγ binding sequence on the CGI-58 promoter. (**b**), Representative immunoblot of PPARγ in liver tissues.


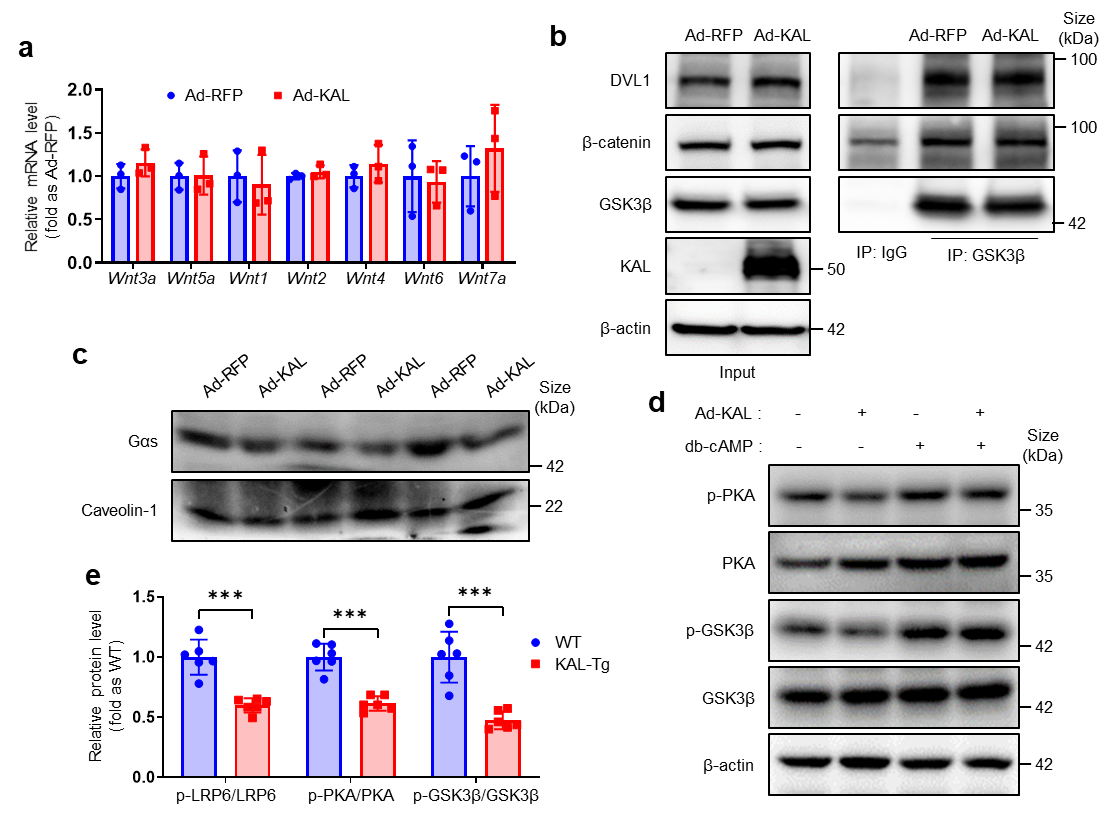


Figure. S6.

**KAL regulates the LRP6/Gαs/PKA/GSK3β signals.** (**a**), mRNA levels of Wnt ligands in primary hepatocytes treated with Ad-KAL or Ad-RFP for 48 h. (**b**), DVL1 and β-catenin immunoblotting after immunoprecipitation (IP) for GSK3β in primary hepatocytes treated with Ad-KAL or Ad-RFP for 48 h, IP for IgG as the negative control. (**c**), Protein levels of Gαs in the membrane of hepatocytes treated with ad-KAL for 48h. (**d**), Protein levels of p-GSK3β in hepatocytes treated with Ad-KAL and db-cAMP for 48 h. (**e**), Quantification of immunoblot in Fig.7D. Data represent the mean ± SD. ****p<0.001*.


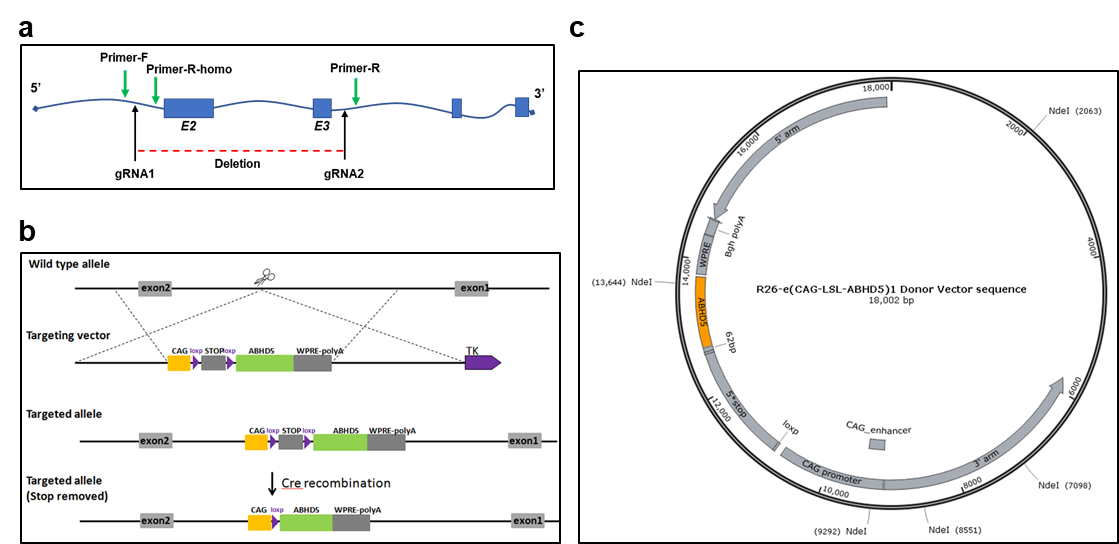


Figure. S7.

**The information for generating the KAL knockout rat strain and CGI-58 transgenic mouse strain.** (a) Schematic diagram of generating the KAL knockout rat strain. (b) Schematic diagram of generating the CGI-58 transgenic mouse strain. (c) Map of CGI-58 homologous recombinant plasmid.


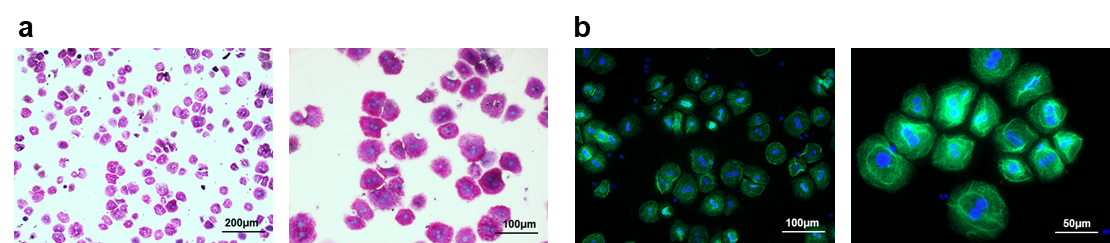


Figure. S8.

**The identification of mouse primary hepatocytes.** (**a**), Periodic acid schiff (PAS) staining in primary hepatocytes. (**b**), CK-18 (green) immunostaining in primary hepatocytes. Images were acquired under a fluorescence microscope.

Table S1.

**Clinical and biochemical characteristics of HLP patients.**

|  | **Control** | **HLP** | **HLP w/o HTG** | **HTG** |  |
| --- | --- | --- | --- | --- | --- |
| N | 221 | 253 | 167 | 86 |  |
| Male | | 112 | 179 (71% of HLP) | 111 | 68 (38% of male HLP) |
| Female | | 109 | 74 (29% of HLP) | 56 | 18 (24% of female HLP) |
| Age (years) | 49 ± 12 | 49 ± 11 | 49 ± 11 | 50 ± 11 |  |
| AST (U/L) | 21.01 ± 5.02 | 21.61 ± 4.78 | 21.18 ± 4.60 | 22.45 ± 5.03^a,b^ |  |
| ALT (U/L) | 18.30 ± 7.15 | 20.88 ± 7.85^a^ | 19.16 ± 7.16 | 24.22 ± 8.10^a,b^ |  |
| TC (mM) | 4.34 ± 0.51 | 5.56 ± 0.88^a^ | 5.60 ± 0.83^a^ | 5.49 ± 0.97^a^ |  |
| TG (mM) | 0.87 ± 0.29 | 1.58 ± 0.87^a^ | 1.11 ± 0.28^a^ | 2.48 ± 0.92^a,b^ |  |
| HDL-C (mM) | 1.30 ± 0.22 | 1.16 ± 0.29^a^ | 1.24 ± 0.29^a^ | 1.01 ± 0.20^a,b^ |  |
| LDL-C (mM) | 2.43 ± 0.45 | 3.52 ± 0.75^a^ | 3.64 ± 0.67^a^ | 3.30 ± 0.86^a,b^ |  |
| Glucose (mM) | 5.05 ± 0.48 | 5.23 ± 0.58^a^ | 5.14 ± 0.52 | 5.41 ± 0.66^a,b^ |  |

Mean ± SD; a: vs Control, *p<0.05*; b: vs HLP w/o HTG, *p<0.05*.

Table S2.

**Clinical and biochemical characteristics of NAFLD patients.**

|  | **Control** | **NAFLD** | ***p* value** |
| --- | --- | --- | --- |
| N | 62 | 44 |  |
| Male | 48 | 35 (80% of NAFLD) |  |
| Female | 14 | 9 (20% of NAFLD) |  |
| Age (years) | 35 ± 10 | 36 ± 11 | *0.469* |
| BMI (kg/m2) | 22.62 ± 2.34 | 25.67 ± 2.08 | *<0.001* |
| AST (U/L) | 20.1 ± 6.95 | 26.52 ± 14.26 | ***0.004*** |
| ALT (U/L) | 19.74 ± 11.54 | 39.93 ± 19.70 | ***<0.001*** |
| TC (mM) | 4.81 ± 0.75 | 5.11 ± 0.83 | *0.067* |
| TG (mM) | 1.09 ± 0.49 | 1.80 ± 0.97 | ***<0.001*** |
| HDL-C (mM) | 1.24 ± 0.27 | 1.13 ± 0.23 | *0.023* |
| LDL-C (mM) | 3.3 ± 0.69 | 3.55 ± 1.34 | *0.215* |
| Glucose (mM) | 5.11 ± 0.63 | 5.53 ± 1.64 | *0.074* |

Mean ± SD, *p* value: NAFLD vs Control.

Table S3.

**NAFLD activity scoring system.**

| **Item** | **Definition** | **Score** |
| --- | --- | --- |
| Steatosis | Percent of hepatocytes with steatosis |  |
|  | < 5% | 0 |
|  | 5%-33% | 1 |
|  | 33%-66% | 2 |
|  | > 66% | 3 |
| Inflammation | Numbers of Inflammatory foci |  |
|  | no foci | 0 |
|  | < 2 foci per 200x field | 1 |
|  | 2-4 foci per 200x field | 2 |
|  | > 4 foci per 200x field | 3 |
| Ballooning |  |  |
|  | None | 0 |
|  | Few balloon cells | 1 |
|  | Many cells/prominent ballooning | 2 |
| Fibrosis Stage |  |  |
|  | None | 0 |
|  | Perisinusoidal or periportal | 1 |
|  | Perisinusoidal and portal/periportal | 2 |
|  | Bridging fibrosis | 3 |
|  | Cirrhosis | 4 |

Table S4.

**Antibodies information.**

| **Antibodies** | **Supplier** | **Catalog number** | **Clone name** | **Application** | **RRID** |
| --- | --- | --- | --- | --- | --- |
| KAL | Abcam | ab187656 | EPR15310 | WB | N/A |
| CGI-58 | Santa Cruz | sc-100468 | 36A | WB, IP | AB_2220720 |
| CGI-58 | Santa Cruz | sc-376931 | E-1 | IF | AB_2868519 |
| TNFα | CST | 3707 | Polyclonal | WB | AB_2240625 |
| NF κB p65 | Abcam | ab16502 | Polyclonal | WB, IP, IF | AB_443394 |
| IL-6 | Abcam | ab208113 | Polyclonal | WB | AB_2927421 |
| α-SMA | Abcam | ab5694 | Polyclonal | WB | AB_2223021 |
| KLF4 | Abcam | ab214666 | EPR20753-25 | WB | N/A |
| CK-18 | Beyotime | AF1285 | Polyclonal | IF | N/A |
| AKT | CST | 4691 | C67E7 | WB | AB_915783 |
| p-AKT (Ser473) | CST | 4060 | D9E | WB | AB_2315049 |
| GLUT2 | Santa Cruz | sc-9117 | H-67 | WB | AB_641068 |
| ATGL | cayman | 10006409 | Polyclonal | WB | AB_10141766 |
| CPT1A | Proteintech | 15184-1-AP | Polyclonal | WB | AB_2084676 |
| FAS | CST | 3180 | C20G5 | WB | AB_2100796 |
| PPARγ | CST | 2435 | C26H12 | WB | AB_2166051 |
| CD36 | Abcam | ab133625 | EPR6573 | WB | AB_2716564 |
| ACC | CST | 3676 | C83B10 | WB | AB_2219397 |
| p-ACC | CST | 3661 | Polyclonal | WB | AB_330337 |
| hsp90 | CST | 4877 | C45G5 | WB | AB_2233307 |
| β-tubulin | Abcam | ab179513 | EPR16774 | WB | N/A |
| α-tubulin | CST | 3873 | DM1A | WB | AB_1904178 |
| GAPDH | Sigma | G8795 | GAPDH-71.1 | WB | AB_1078991 |
| histone | Abcam | ab1791 | Polyclonal | WB | AB_302613 |
| β-actin | Sigma | A5441 | AC-15 | WB | AB_476744 |
| Anti-rabbit | Vector Laboratory | PI-1000 |  | WB | AB_2336198 |
| Anti-mouse | Vector Laboratory | PI-2000 |  | WB | AB_2336177 |
| Anti-rabbit 488 | Thermo Fisher | A32731 |  | IF | AB_2633280 |
| Anti-mouse 594 | Thermo Fisher | A-11032 |  | IF | AB_2534091 |

Table S5.

**Interference sequence and primer sequence.**

| **Name** | **Sequence** |
| --- | --- |
| m-si-*Abhd5* | CCAGAAGAATTCAACCAGA |
| m-si-*Klf4* | GCAGCTTGCAGCAGTAACA |
| h-*SERPINA4*-F | GCATCTTCCCAAGTTCTCCATT |
| h-*SERPINA4*-R | ATGCCGGATAAGTCAGCCCA |
| Rat-*Serpina4*-F | AGGAGGAATTGTTCCATCTGAGAG |
| Rat-*Serpina4*-R | GGTCTGGCTGACTGTGAGAG |
| m-*Tnf* -F | CCCTCACACTCAGATCATCTTCT |
| m-*Tnf* -R | GCTACGACGTGGGCTACAG |
| Rat-*Tnf* -F | CTTCTCATTCCTGCTCGTGG |
| Rat-*Tnf* -R | CTCCGCTTGGTGGTTTGCTA |
| m-*Col1a1*-F | CGATGGATTCCCGTTCGAGT |
| m-*Col1a1*-R | GAGGCCTCGGTGGACATTAG |
| Rat-*Col1a1*-F | GAAACTTTGCTTCCCAGA |
| Rat-*Col1a1*-R | ATCATCTCCGTTCTTGCC |
| m-*Acta2*-F | GTCCCAGACATCAGGGAGTAA |
| m-*Acta2*-R | TCGGATACTTCAGCGTCAGGA |
| Rat-*Acta2*-F | TGGAAAAGATCTGGCACCAC |
| Rat-*Acta2*-R | TCCGTTAGCAAGGTCGGATG |
| m-*Abhd5*-F | TGGGGTTTTCCTGAGCGAC |
| m-*Abhd5*-R | GGTTAAAGGGAGTCAATGCTGC |
| m-*Pnpla2*-F | ACGCCACTCACATCTACGGA |
| m-*Pnpla2*-R | CAATCAGCAGGCAGGGTCTT |
| m-*Pparg*-F | TGCGGAAGCCCTTTGGTGACT |
| m-*Pparg*-R | ATGTCCTCGATGGGCTTCACGTTC |
| m-*Apob*-F | TCACCATTTGCCCTCAACCTAA |
| m- *Apob*-R | GAAGGCTCTTTGGAAGTGTAAAC |
| m-*Mttp*-F | AACTCCTACGAGCCCTCCTT |
| m-*Mttp*-R | AGTCCTCCCAGGATCAGCTT |
| m-*Actb*-F | GGCTGTATTCCCCTCCATCG |
| m-*Actb*-R | CCAGTTGGTAACAATGCCATGT |
| Rat-*Actb*-F | GAGAGGGAAATCGTGCGTGA |
| Rat-*Actb*-R | CAGGGAGGAAGAGGATGCGG |
