# Supplementary material for: Elevated Kallistatin promotes the occurrence and progression of non-alcoholic fatty liver disease
Source: Signal Transduct Target Ther. 2024 Mar 12;9:66. doi: 10.1038/s41392-024-01781-9 (PMC10933339; doi:10.1038/s41392-024-01781-9)

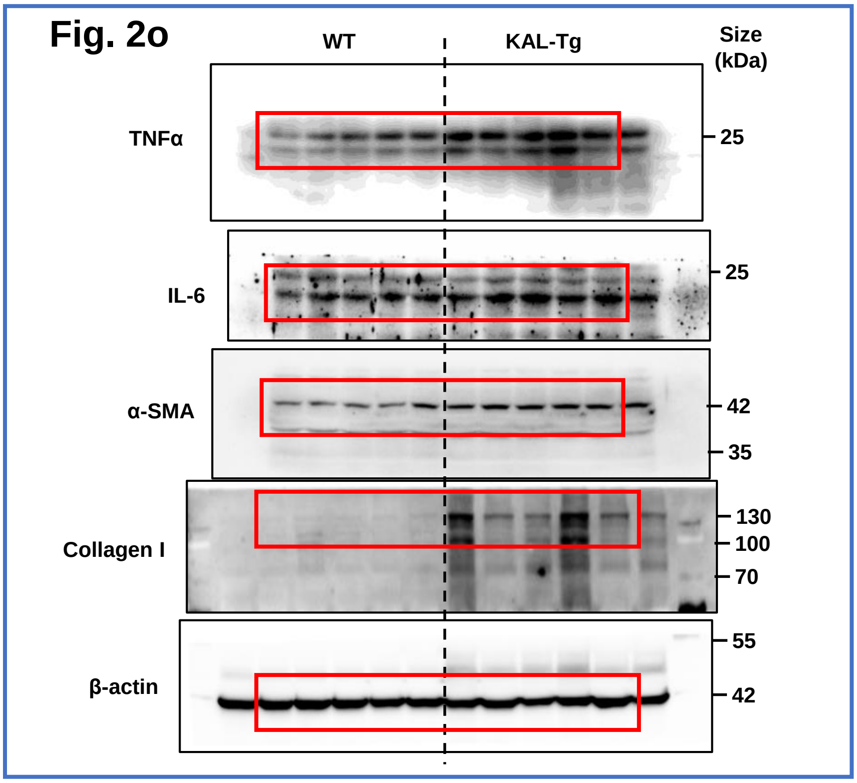
Fig. 2o
WT
KAL-Tg
Size (kDa)
TNFα
25
IL-6
25
α-SMA
42
35
Collagen I
130
100
70
β-actin
55
42

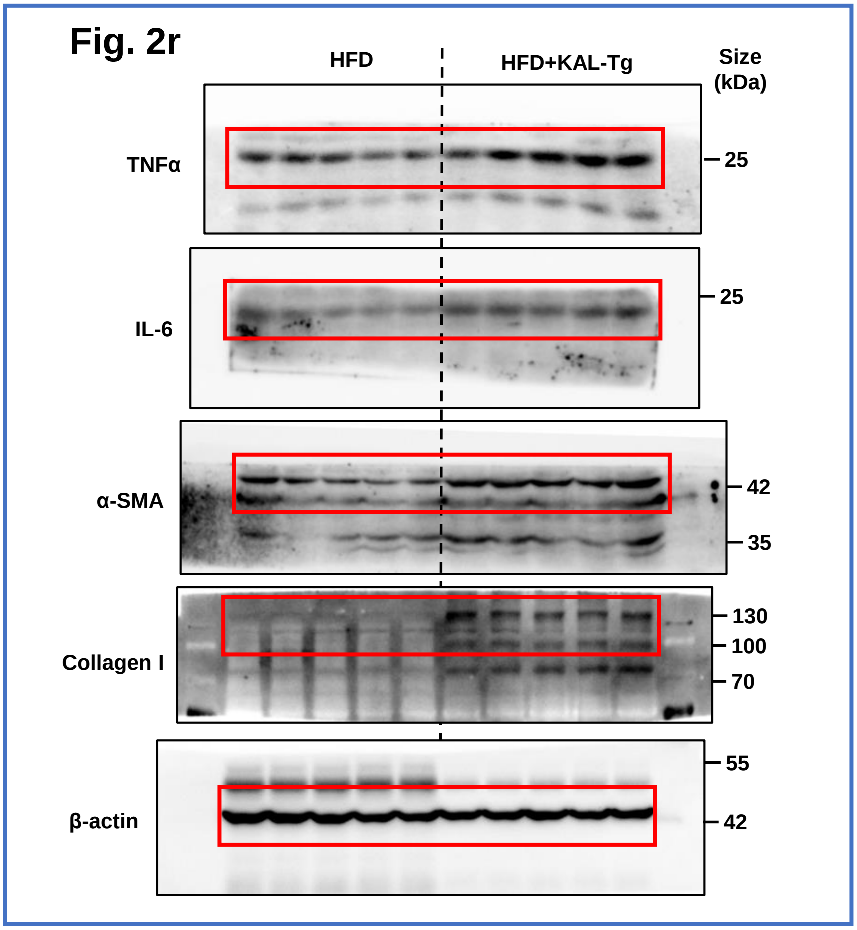
Fig. 2r
HFD
HFD+KAL-Tg
Size (kDa)
TNFα
25
IL-6
25
α-SMA
42
35
Collagen I
130
100
70
β-actin
55
42

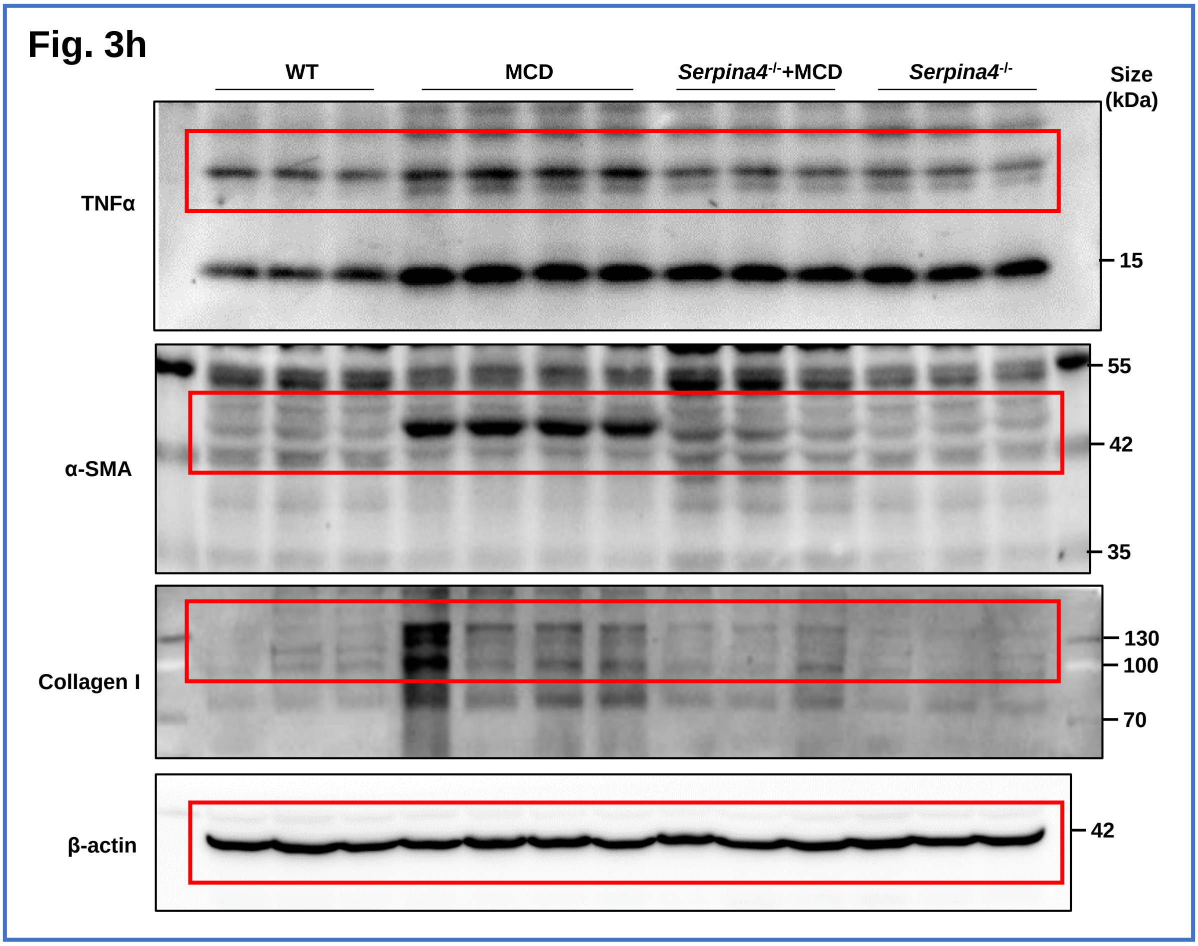
Fig. 3h
WT
MCD
Serpina4-/-+MCD
Serpina4-/-
Size (kDa)
TNFα
15
α-SMA
55
42
35
Collagen I
130
100
70
β-actin
42

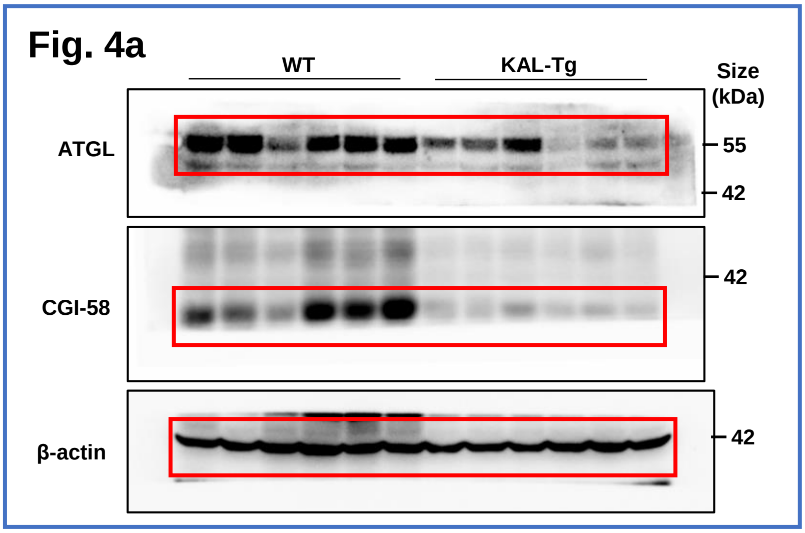
Fig. 4a
WT
KAL-Tg
Size (kDa)
ATGL
55
42
CGI-58
42
β-actin
42

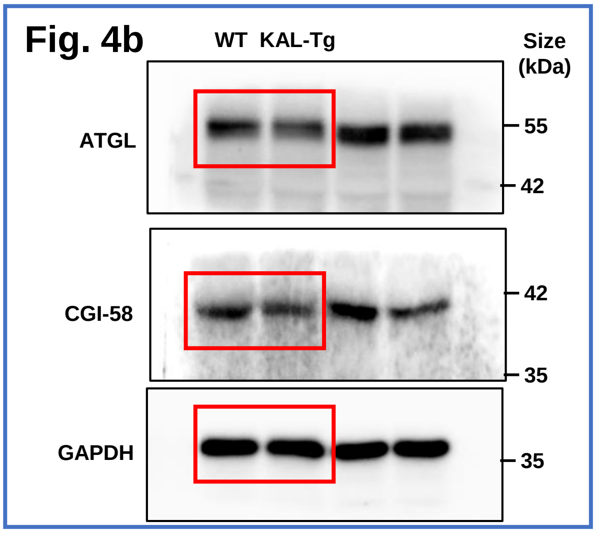
Fig. 4b
WT
KAL-Tg
Size (kDa)
ATGL
55
42
CGI-58
42
35
GAPDH
35

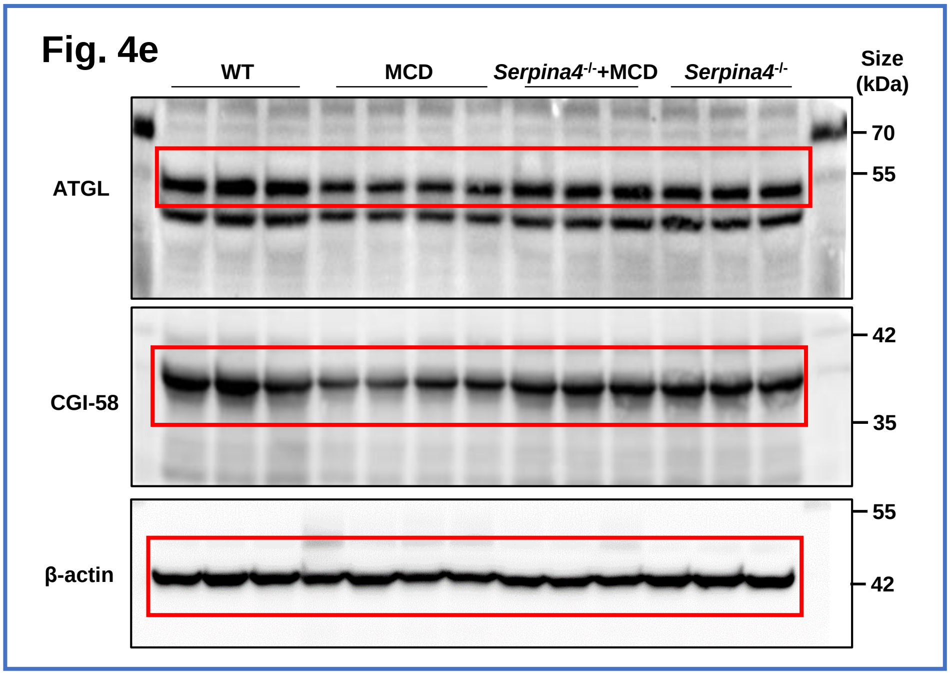
Fig. 4e
WT
MCD
Serpina4-/-+MCD
Serpina4-/-
Size (kDa)
ATGL
70
55
CGI-58
42
35
β-actin
55
42

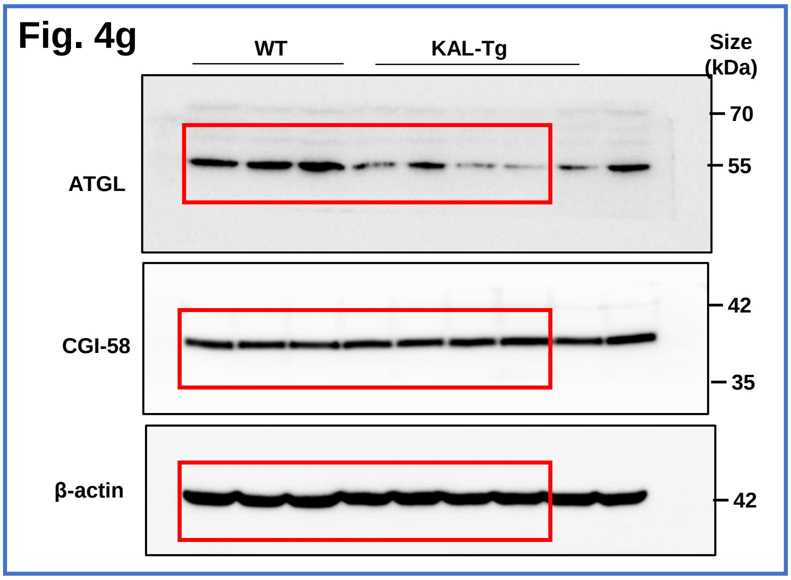
Fig. 4g
WT
KAL-Tg
Size (kDa)
ATGL
70
55
CGI-58
42
35
β-actin
42

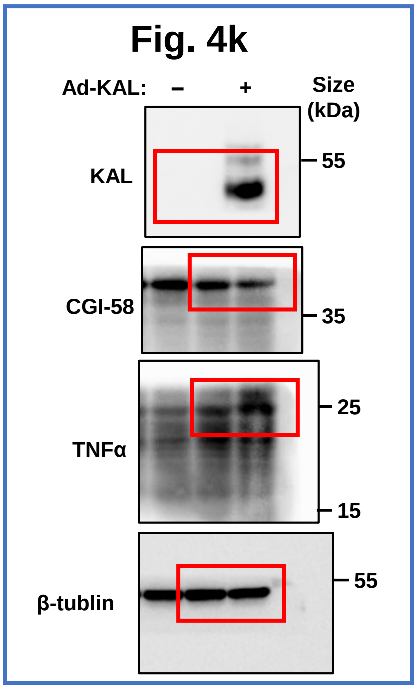
Fig. 4k
Ad-KAL: – +
Size (kDa)
KAL
55
CGI-58
35
TNFα
25
15
β-tublin
55

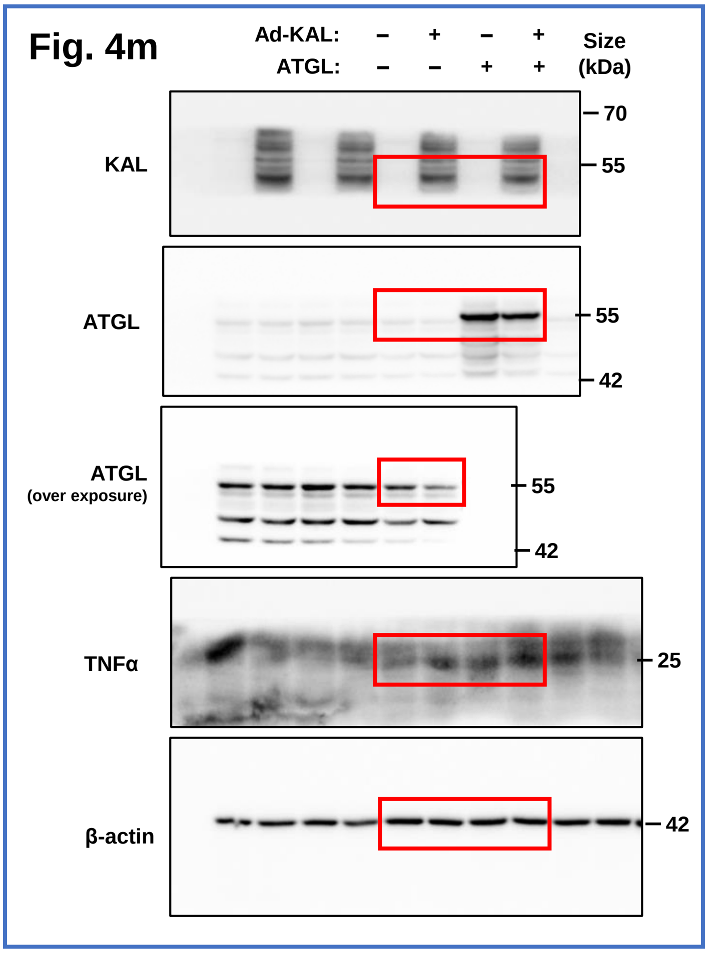
Fig. 4m
Ad-KAL: – + – +
ATGL: – – + +
Size (kDa)
KAL
70
55
ATGL
55
42
ATGL (over exposure)
55
42
TNFα
25
β-actin
42

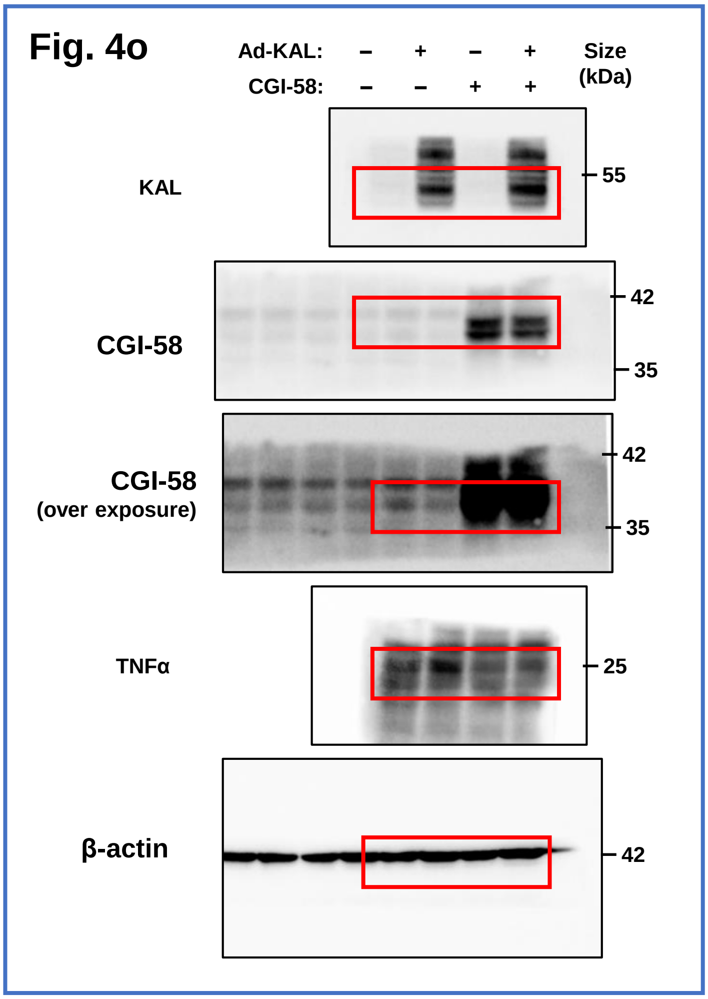
Fig. 4o
Ad-KAL: – + – +
CGI-58: – – + +
Size (kDa)
KAL
55
CGI-58
42
35
CGI-58 (over exposure)
42
35
TNFα
25
β-actin
42

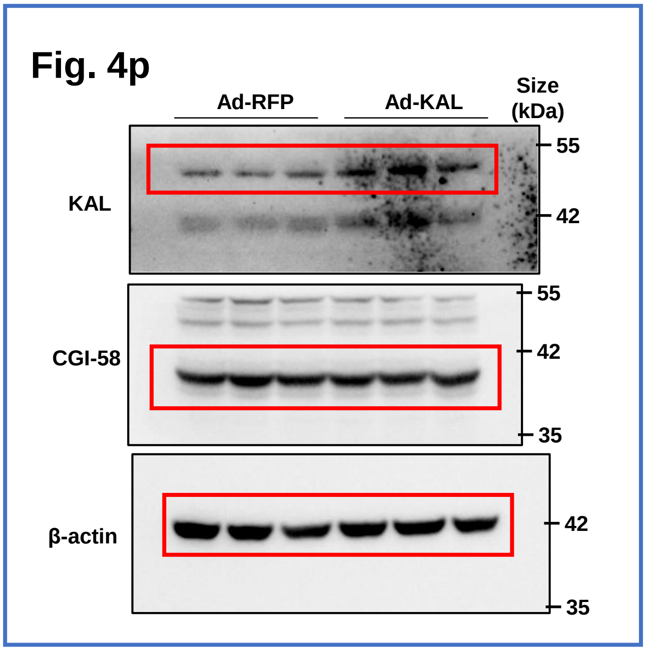
Fig. 4p
Ad-RFP
Ad-KAL
Size (kDa)
KAL
55
42
CGI-58
55
42
35
β-actin
42
35

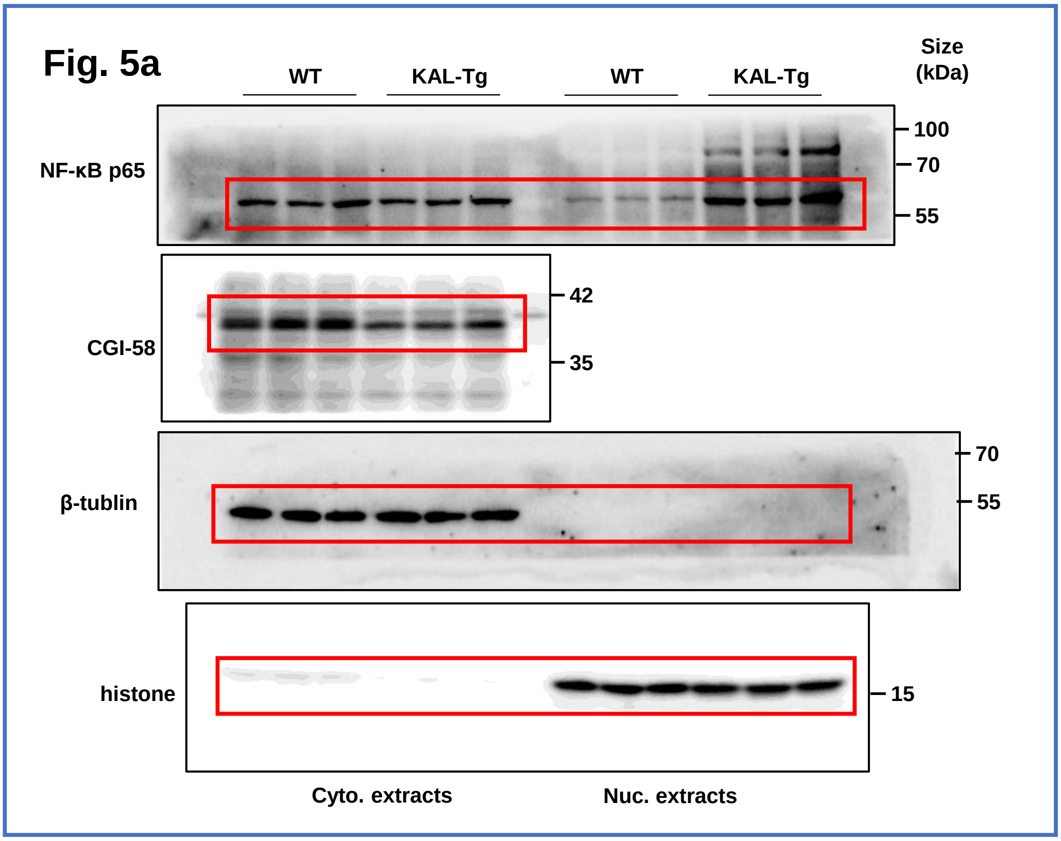

Fig. 5a
WT
KAL-Tg
WT
KAL-Tg
Size (kDa)
NF-κB p65
100
70
55
CGI-58
42
35
β-tublin
70
55
histone
15
Cyto. extracts
Nuc. extracts

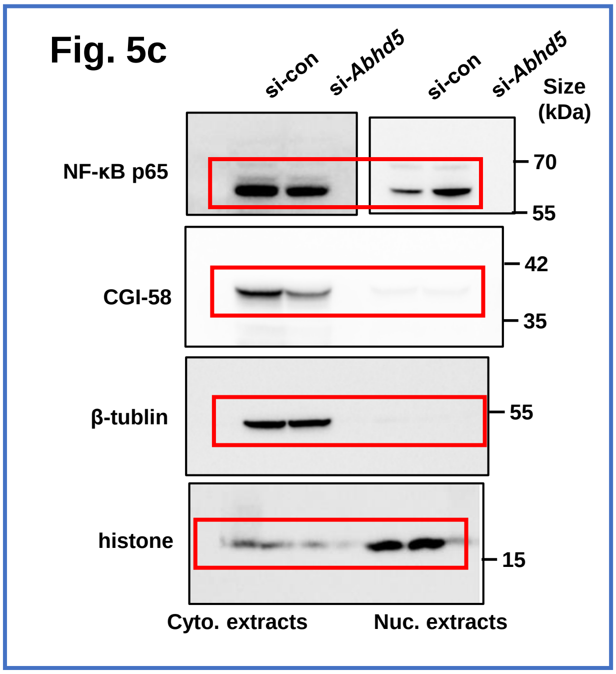

Fig. 5c
si-con
si-Abhd5
si-con
si-Abhd5
Size (kDa)
NF-κB p65
70
55
CGI-58
42
35
β-tublin
55
histone
15
Cyto. extracts
Nuc. extracts

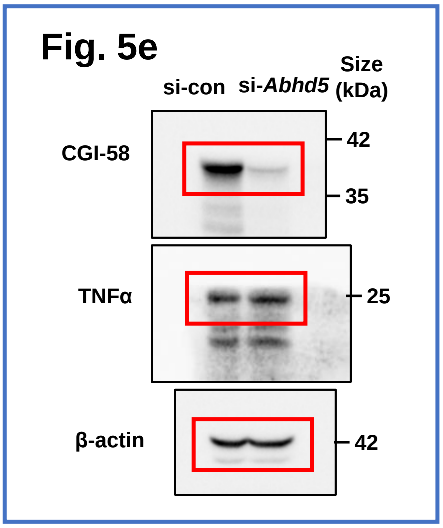

Fig. 5e
si-con
si-Abhd5
Size (kDa)
CGI-58
42
35
TNFα
25
β-actin
42

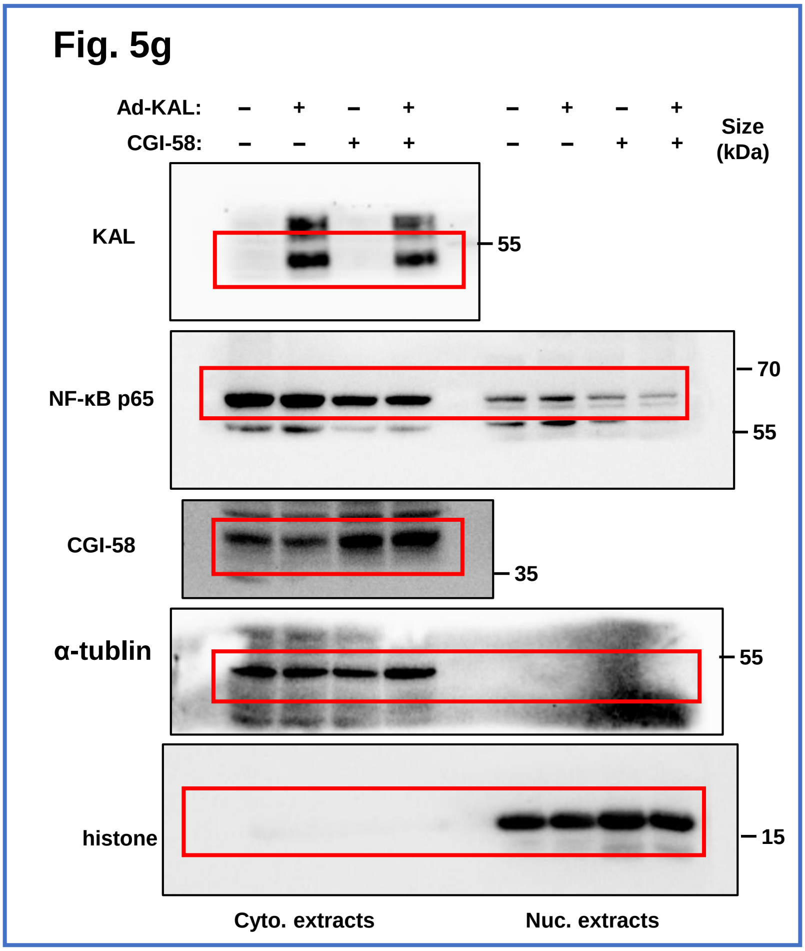
Fig. 5g
Ad-KAL: – + – + – + – +
CGI-58: – – + + – – + +
Size
(kDa)
KAL
55
NF-κB p65
70
55
CGI-58
35
α-tublin
55
histone
15
Cyto. extracts
Nuc. extracts

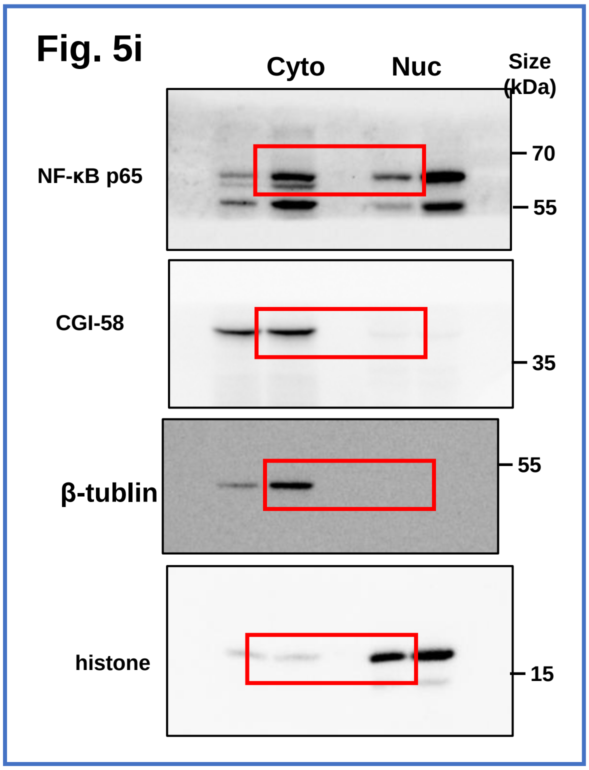
Fig. 5i
Cyto
Nuc
Size
(kDa)
NF-κB p65
70
55
CGI-58
35
β-tublin
55
histone
15

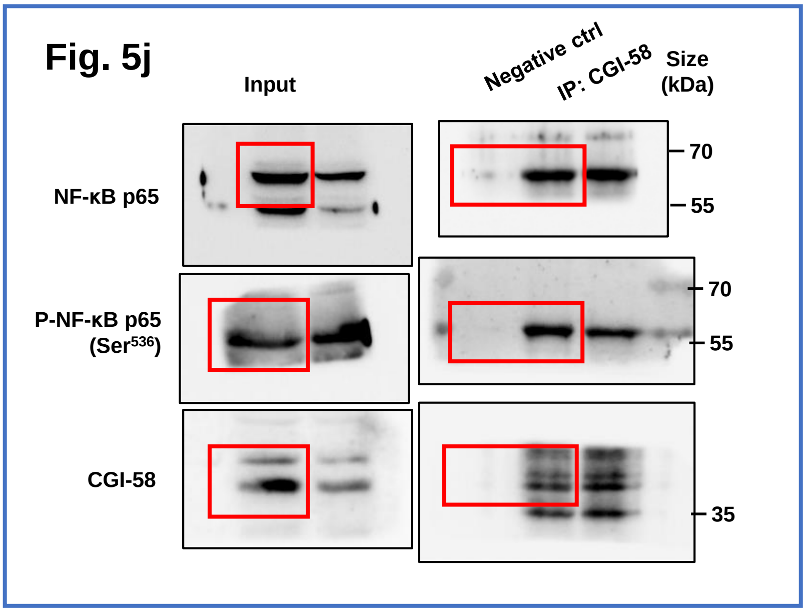
Fig. 5j
Input
Negative ctrl
IP: CGI-58
Size
(kDa)
NF-κB p65
70
55
P-NF-κB p65
(Ser536)
70
55
CGI-58
35

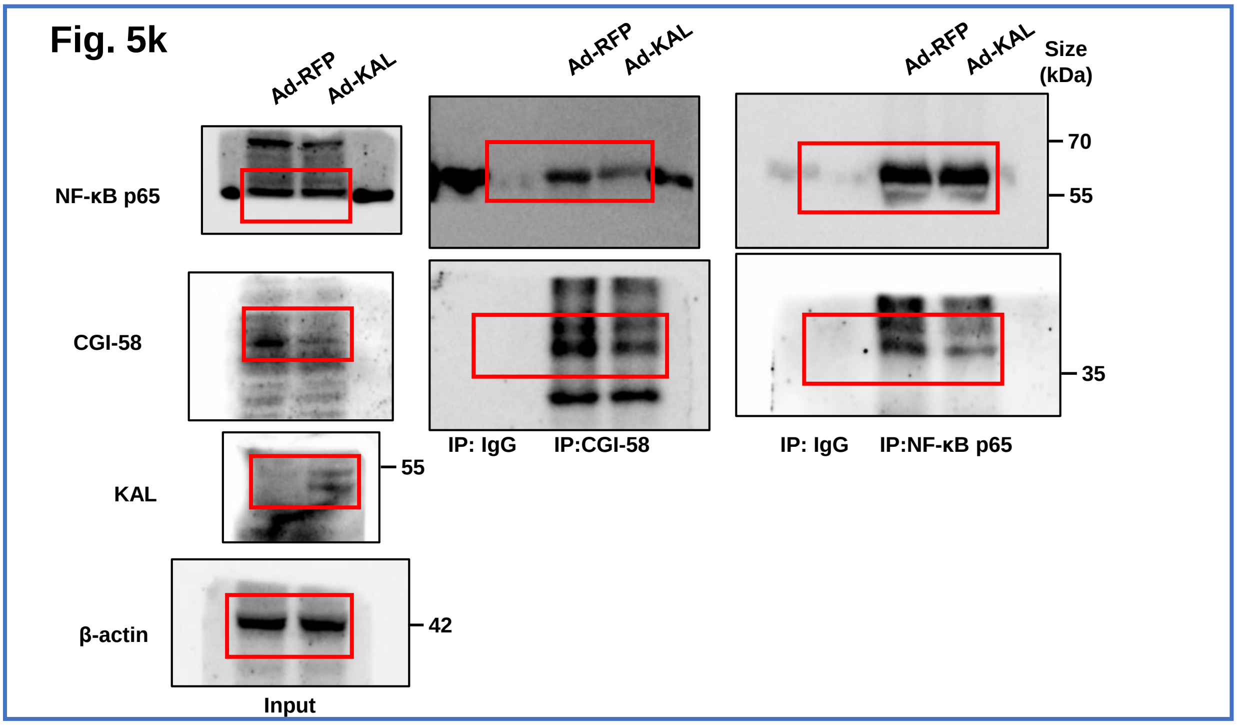
Fig. 5k
Ad-RFP
Ad-KAL
Ad-RFP
Ad-KAL
Ad-RFP
Ad-KAL
Size (kDa)
NF-κB p65
CGI-58
KAL
β-actin
70
55
35
55
42
IP: IgG
IP:CGI-58
IP: IgG
IP:NF-κB p65
Input

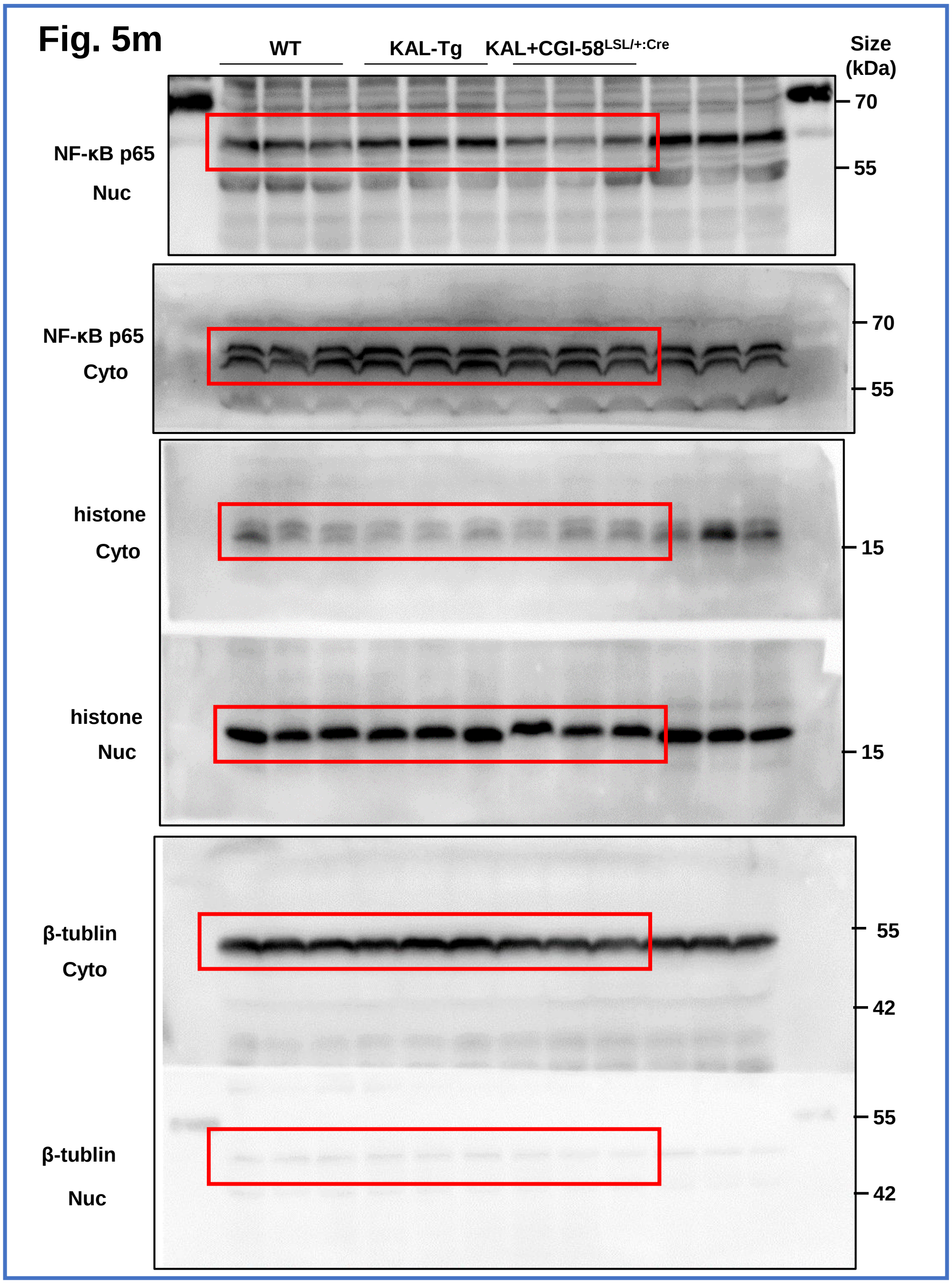
Fig. 5m
WT
KAL-Tg
KAL+CGI-58LSL/+:Cre
Size (kDa)
NF-κB p65 Nuc
NF-κB p65 Cyto
histone Cyto
histone Nuc
β-tublin Cyto
β-tublin Nuc
70
55
70
55
15
15
55
42
55
42

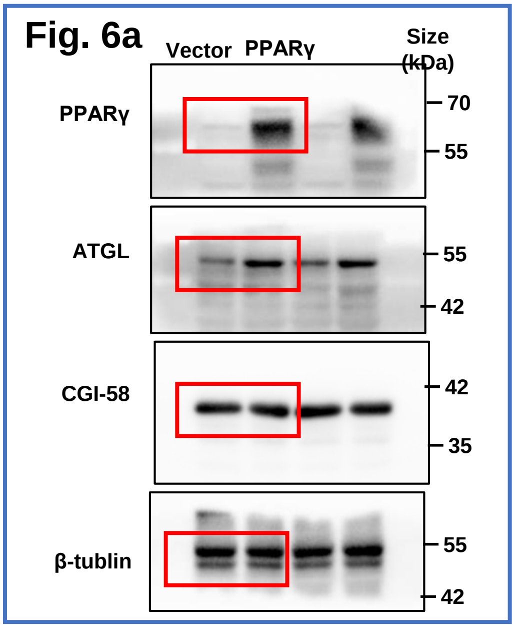
Fig. 6a
Vector
PPARγ
Size (kDa)
PPARγ
70
55
ATGL
55
42
CGI-58
42
35
β-tublin
55
42

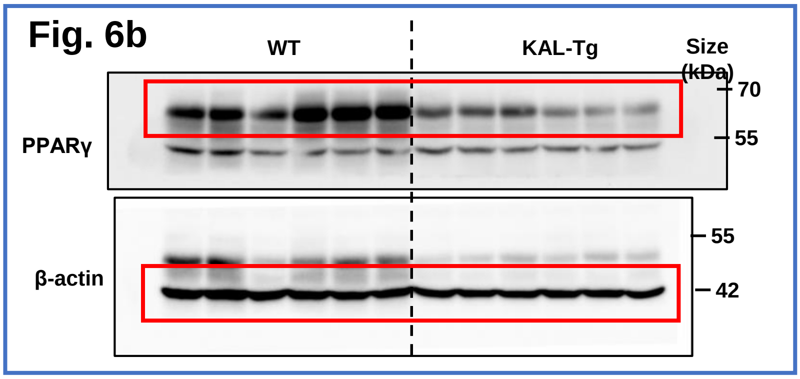
Fig. 6b
WT
KAL-Tg
Size (kDa)
PPARγ
70
55
β-actin
55
42

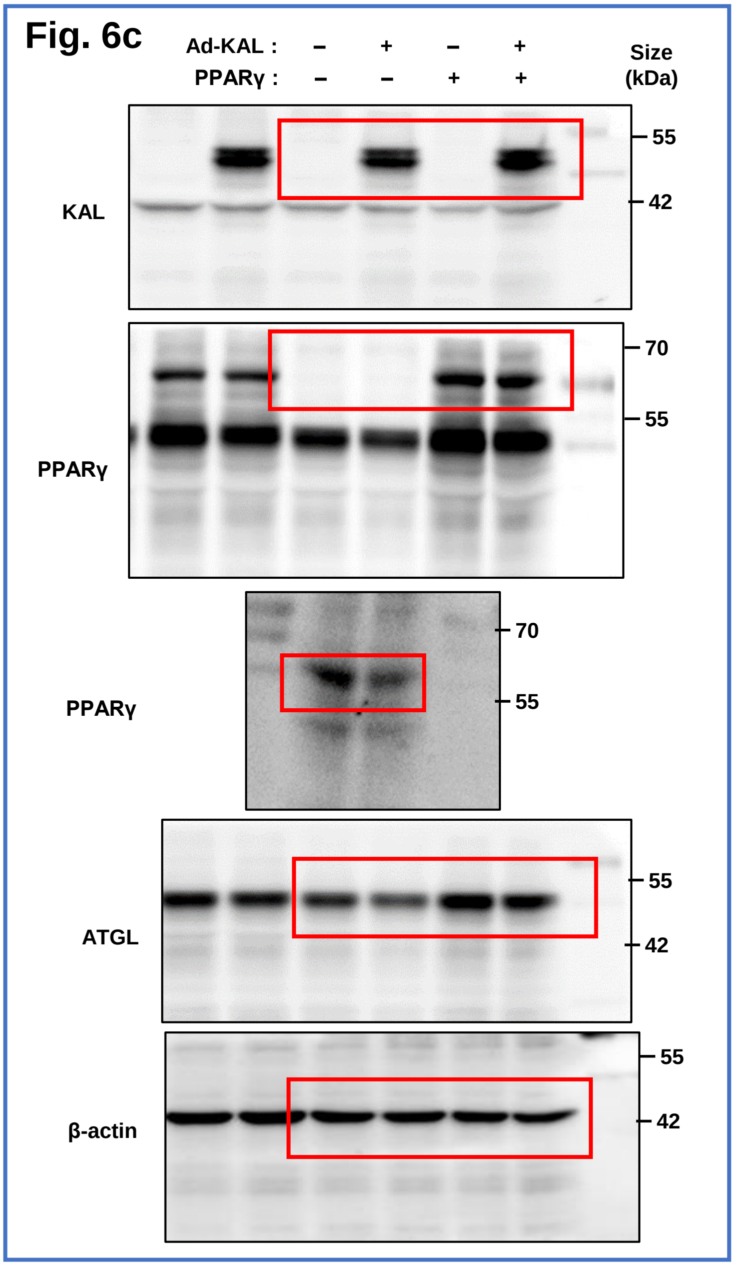
Fig. 6c
Ad-KAL : − + − +
PPARγ : − − + +
Size (kDa)
KAL
55
42
PPARγ
70
55
PPARγ
70
55
ATGL
55
42
β-actin
55
42

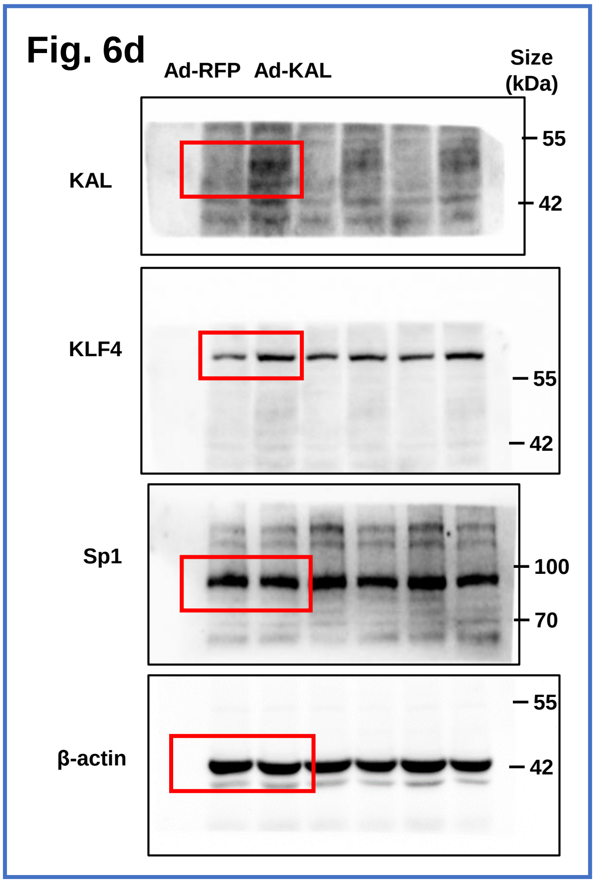
Fig. 6d
Ad-RFP
Ad-KAL
Size (kDa)
KAL
55
42
KLF4
55
42
Sp1
100
70
β-actin
55
42

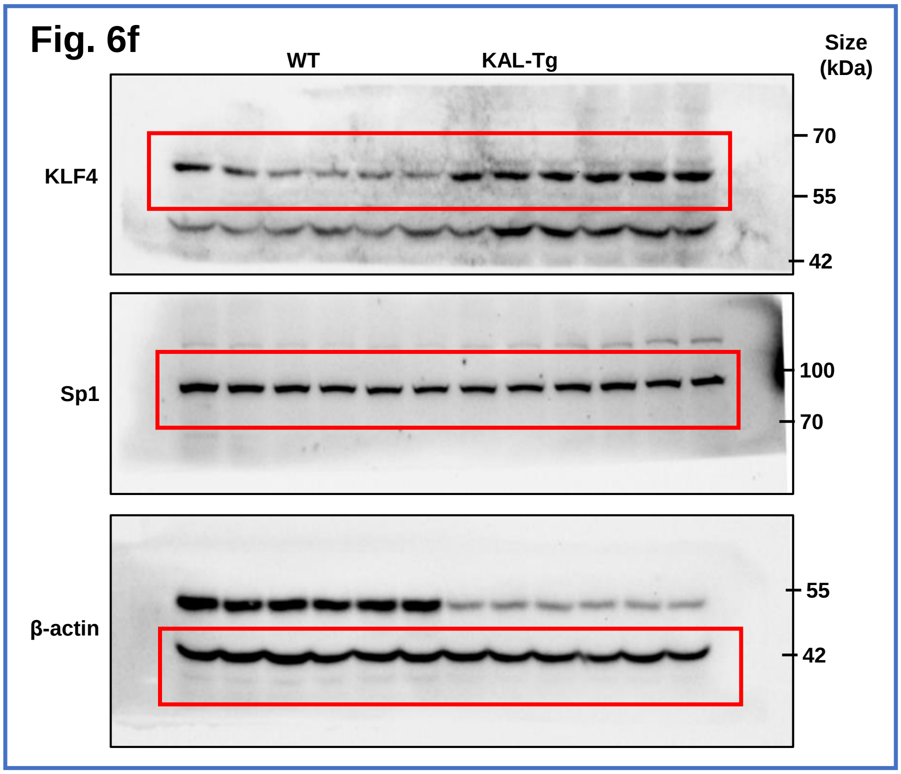
Fig. 6f
WT
KAL-Tg
Size (kDa)
KLF4
70
55
42
Sp1
100
70
β-actin
55
42

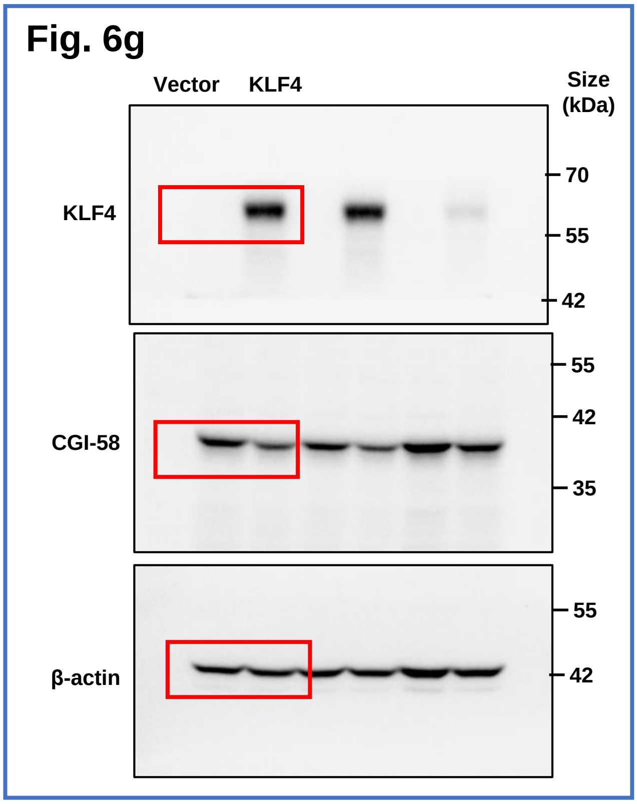
Fig. 6g
Vector
KLF4
Size (kDa)
KLF4
70
55
42
CGI-58
55
42
35
β-actin
55
42

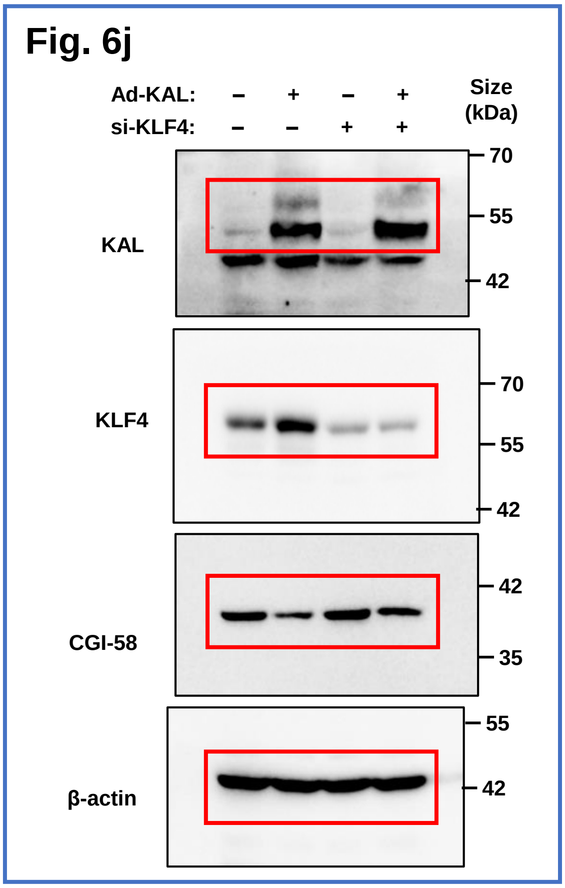
Fig. 6j
Ad-KAL: − + − +
si-KLF4: − − + +
Size (kDa)
KAL
70
55
42
KLF4
70
55
42
CGI-58
42
35
β-actin
55
42

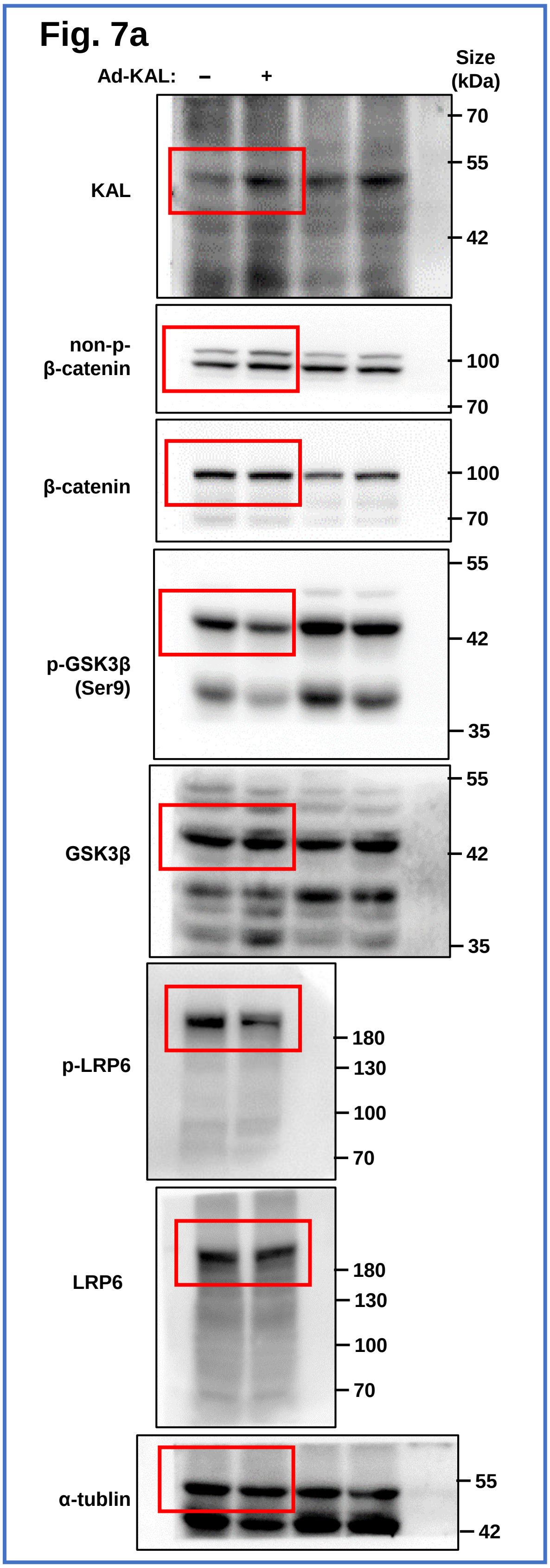
Fig. 7a
Ad-KAL: – +
Size (kDa)
KAL
70
55
42
non-p-β-catenin
100
70
β-catenin
100
70
p-GSK3β (Ser9)
55
42
35
GSK3β
55
42
35
p-LRP6
180
130
100
70
LRP6
180
130
100
70
α-tublin
55
42

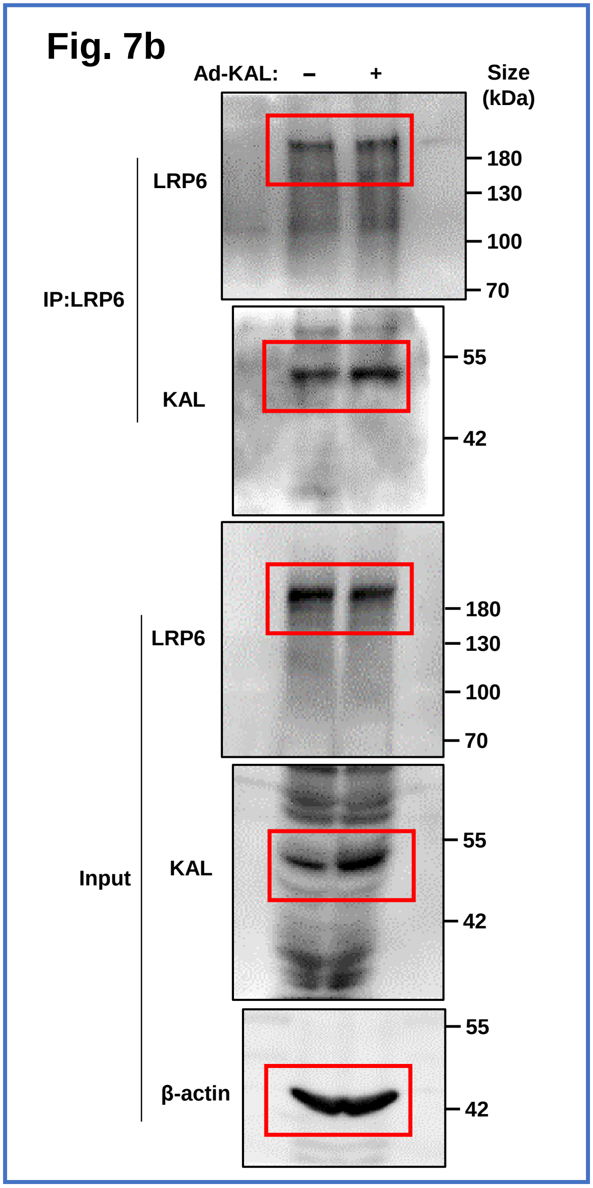
Fig. 7b
Ad-KAL: – +
Size (kDa)
IP:LRP6
LRP6
180
130
100
70
KAL
55
42
Input
LRP6
180
130
100
70
KAL
55
42
β-actin
55
42

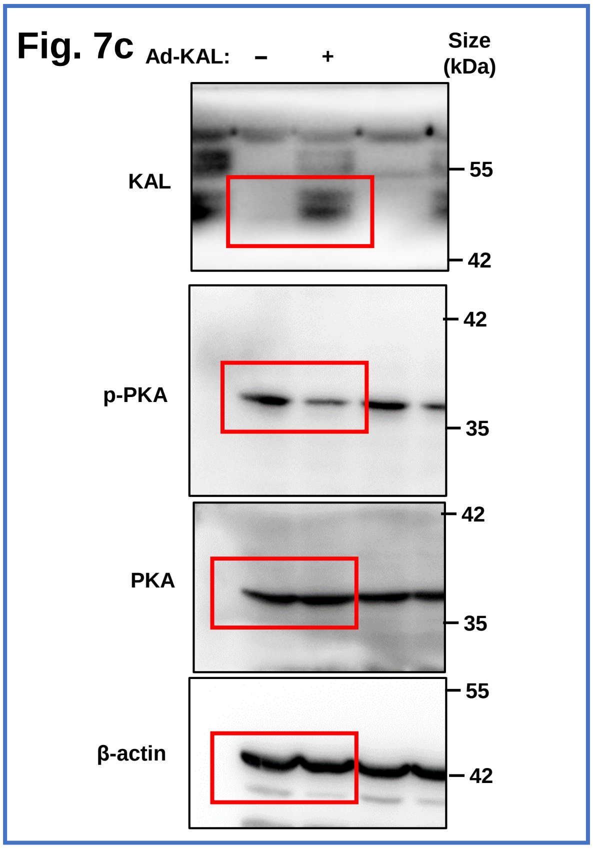
Fig. 7c
Ad-KAL: – +
Size (kDa)
KAL
55
42
p-PKA
42
35
PKA
42
35
β-actin
55
42

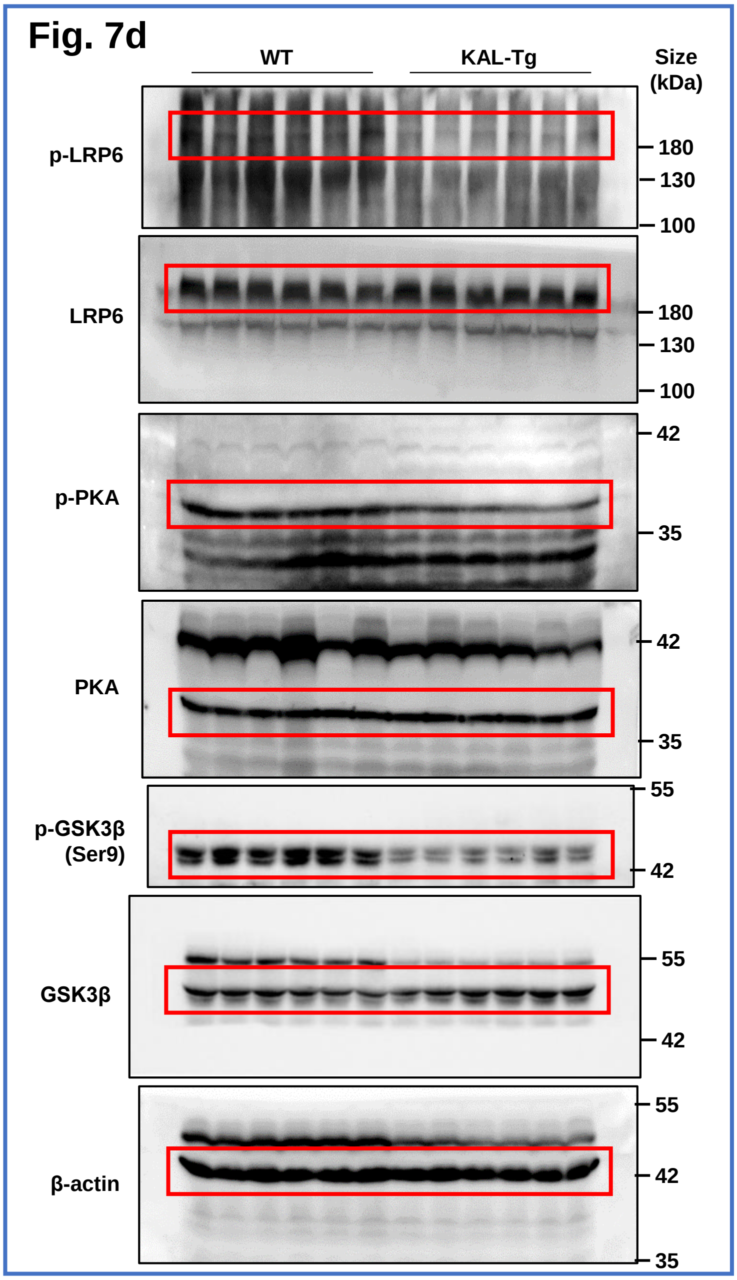
Fig. 7d
WT
KAL-Tg
Size (kDa)
p-LRP6
180
130
100
LRP6
180
130
100
p-PKA
42
35
PKA
42
35
p-GSK3β (Ser9)
55
42
GSK3β
55
42
β-actin
55
42
35

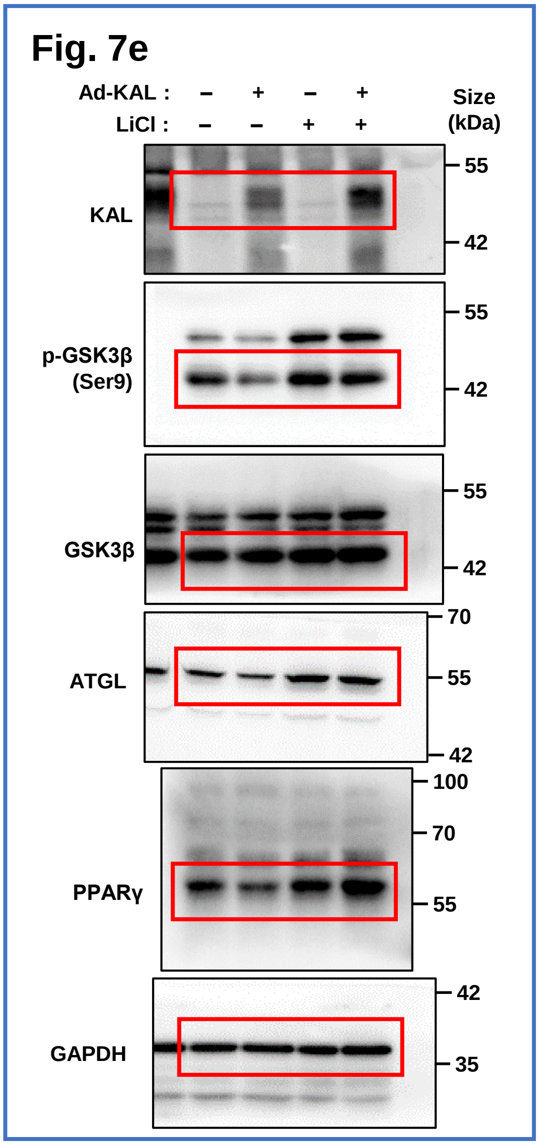
Fig. 7e
Ad-KAL : – + – +
LiCl : – – + +
Size (kDa)
KAL
55
42
p-GSK3β (Ser9)
55
42
GSK3β
55
42
ATGL
70
55
42
PPARγ
100
70
55
GAPDH
42
35

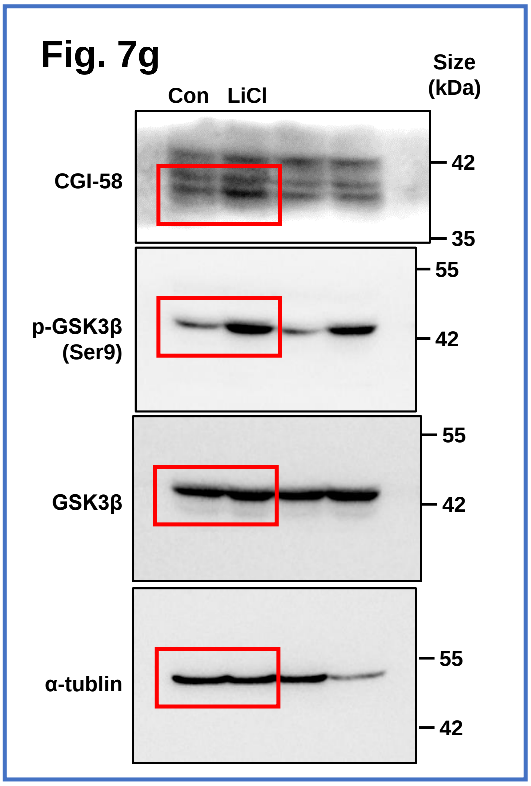
Fig. 7g
Con LiCl
Size (kDa)
CGI-58
42
35
p-GSK3β (Ser9)
55
42
GSK3β
55
42
α-tublin
55
42

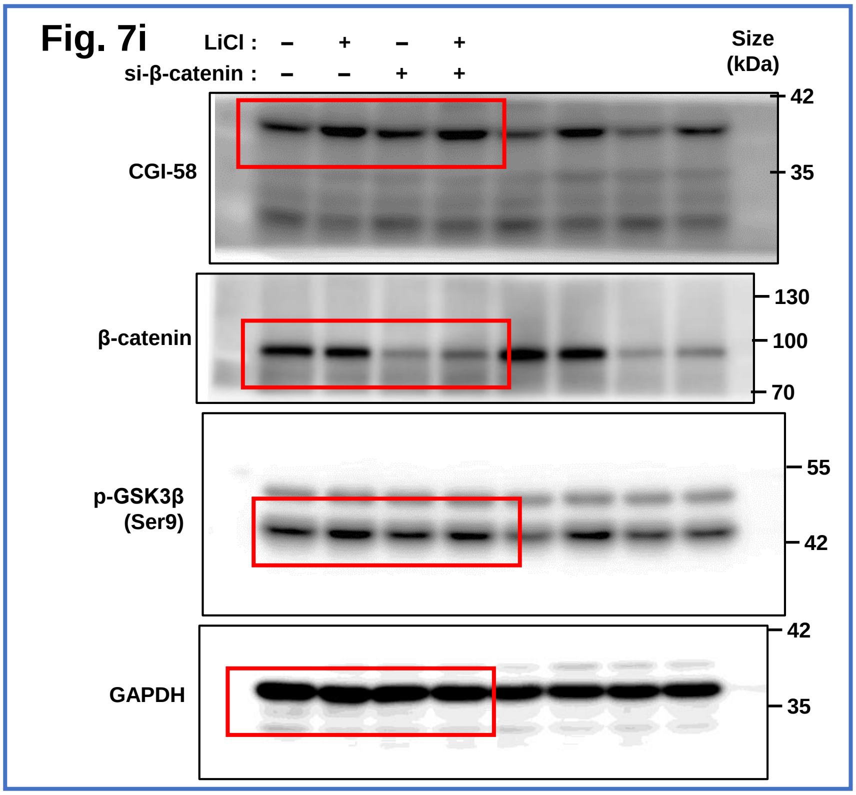
Fig. 7i
LiCl : – + – +
si-β-catenin : – – + +
Size (kDa)
CGI-58
42
35
β-catenin
130
100
70
p-GSK3β (Ser9)
55
42
GAPDH
42
35

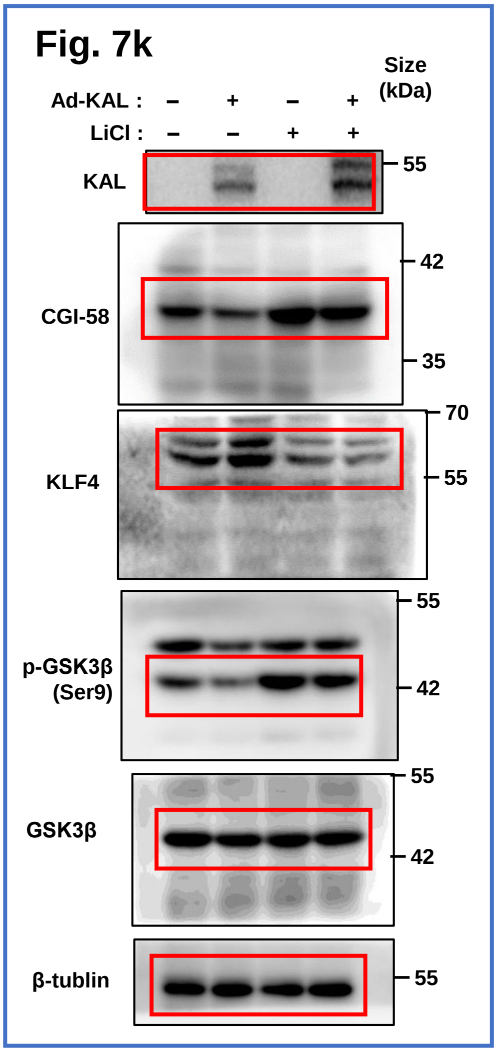
Fig. 7k
Size (kDa)
Ad-KAL : – + – +
LiCl : – – + +
KAL
55
CGI-58
42
35
KLF4
70
55
p-GSK3β (Ser9)
55
42
GSK3β
55
42
β-tublin
55

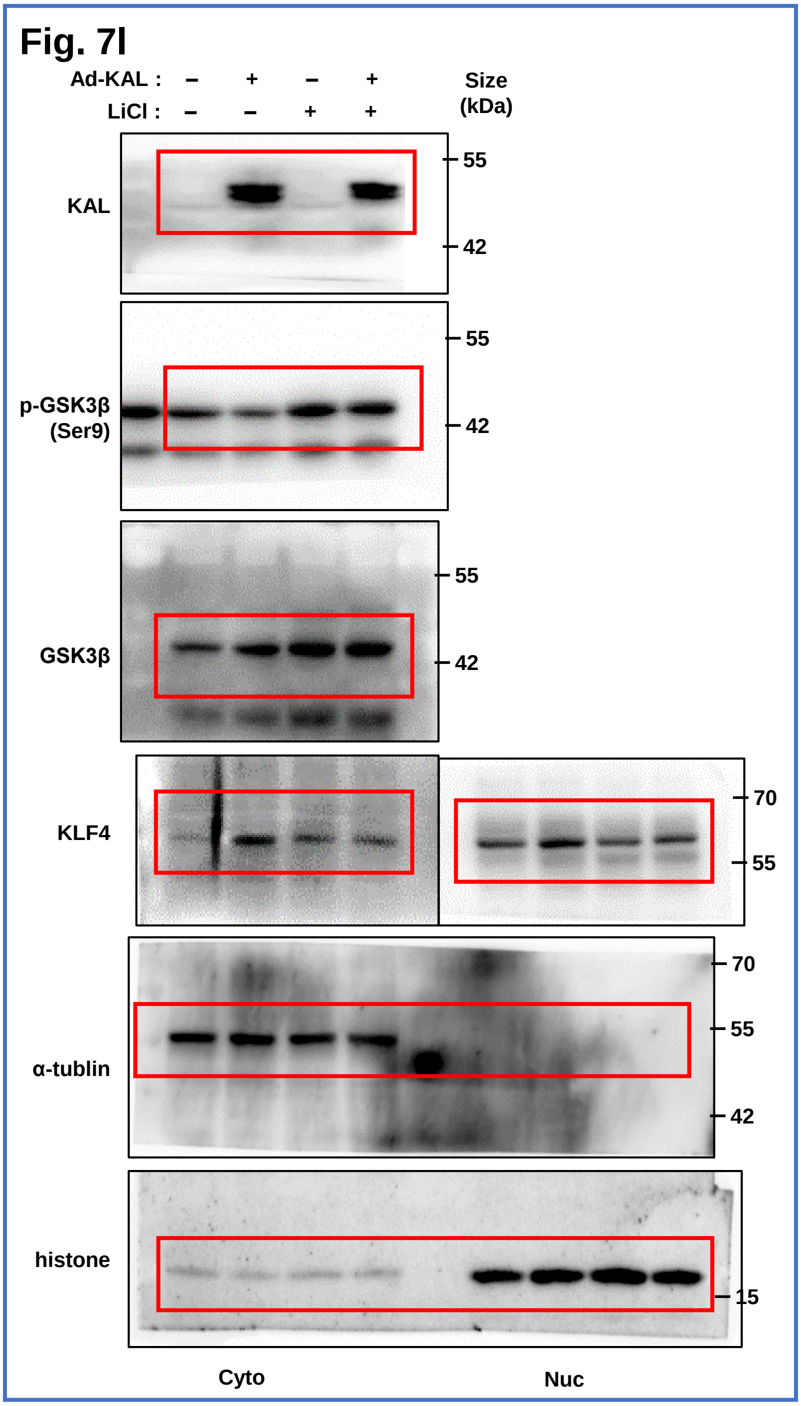
Fig. 7l
Ad-KAL : – + – +
LiCl : – – + +
Size (kDa)
KAL
55
42
p-GSK3β (Ser9)
55
42
GSK3β
55
42
KLF4
70
55
α-tublin
70
55
42
histone
15
Cyto
Nuc

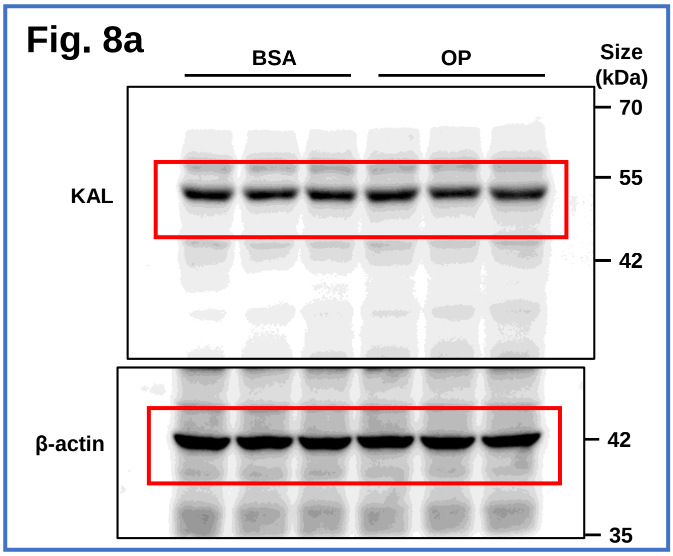
Fig. 8a
BSA
OP
Size
(kDa)
70
55
42
KAL
β-actin
42
35

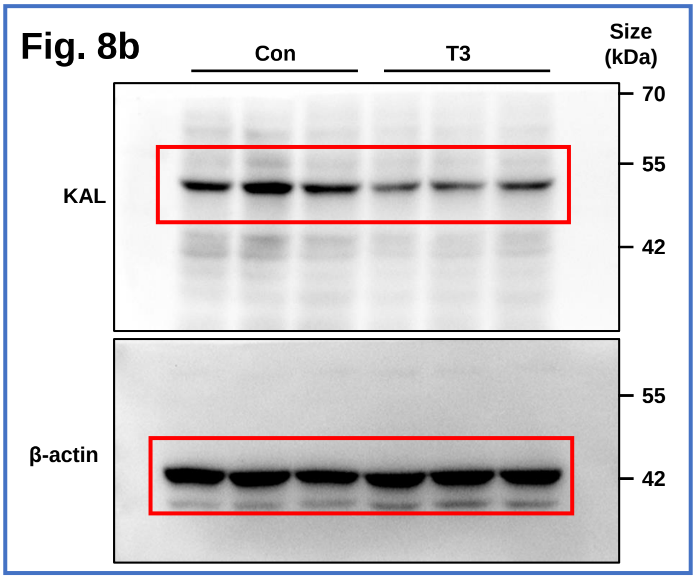
Fig. 8b
Con
T3
Size
(kDa)
70
55
42
KAL
β-actin
55
42

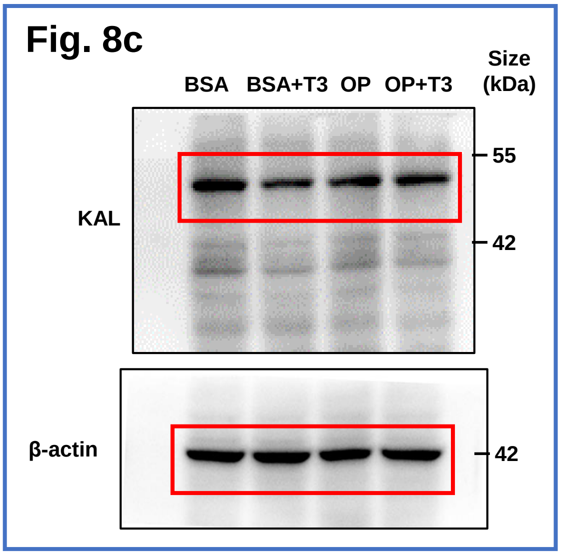
Fig. 8c
BSA
BSA+T3
OP
OP+T3
Size
(kDa)
55
42
KAL
β-actin
42

Fig. 8i

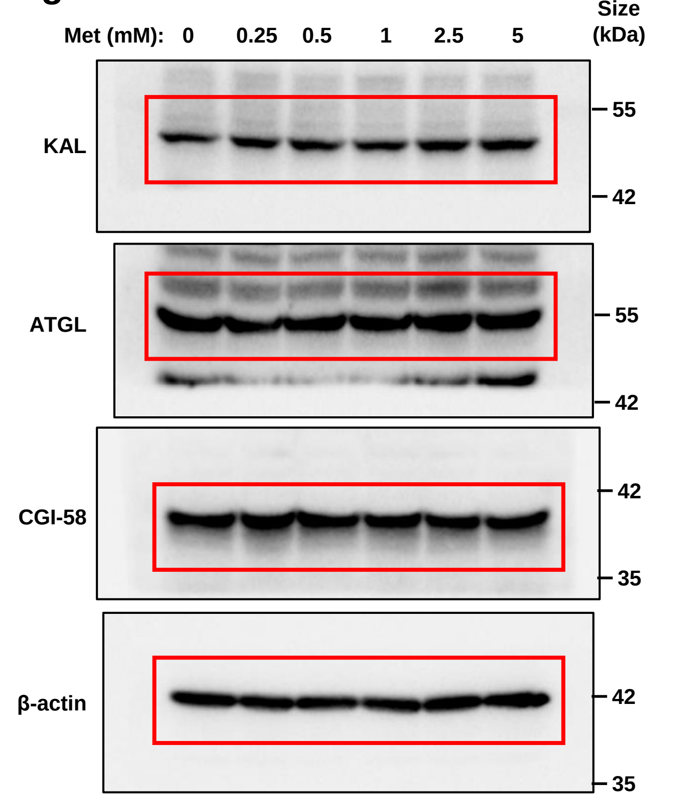

Fig. 8j

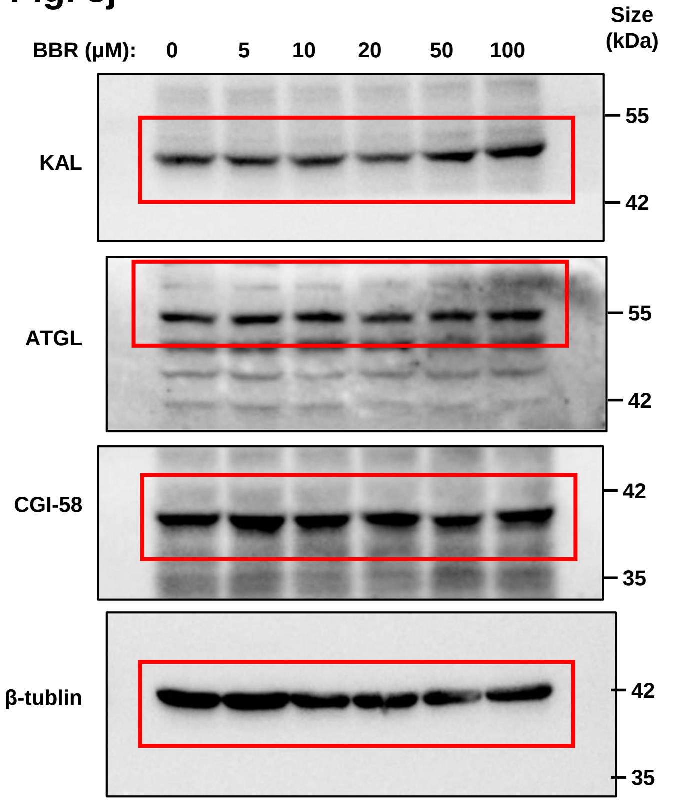

Fig. 8k

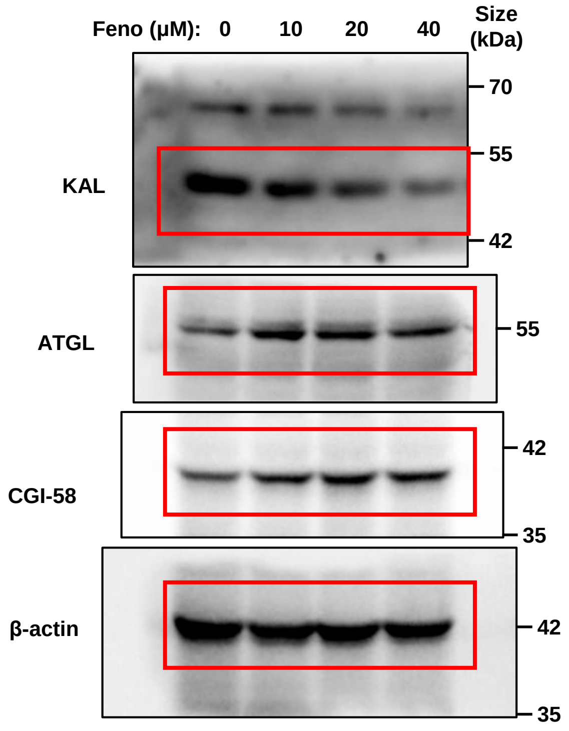

Supplement: Supplementary file 2 — Unmodified gels [file 41392_2024_1781_MOESM2_ESM.pdf]
